# Review and Comparison of Cancer Biomarker Trends in Urine as a Basis for New Diagnostic Pathways

**DOI:** 10.3390/cancers11091244

**Published:** 2019-08-25

**Authors:** Carmen Bax, Beatrice Julia Lotesoriere, Selena Sironi, Laura Capelli

**Affiliations:** Politecnico di Milano, Department of Chemistry, Materials and Chemical Engineering “Giulio Natta”, piazza Leonardo da Vinci 32, 20133 Milan, Italy

**Keywords:** urine metabolites, odor threshold, cancer diagnosis, volatile organic compounds (VOC), volatiles, olfaction

## Abstract

Cancer is one of the major causes of mortality worldwide and its already large burden is projected to increase significantly in the near future with a predicted 22 million new cancer cases and 13 million cancer-related deaths occurring annually by 2030. Unfortunately, current procedures for diagnosis are characterized by low diagnostic accuracies. Given the proved correlation between cancer presence and alterations of biological fluid composition, many researchers suggested their characterization to improve cancer detection at early stages. This paper reviews the information that can be found in the scientific literature, regarding the correlation of different cancer forms with the presence of specific metabolites in human urine, in a schematic and easily interpretable form, because of the huge amount of relevant literature. The originality of this paper relies on the attempt to point out the odor properties of such metabolites, and thus to highlight the correlation between urine odor alterations and cancer presence, which is proven by recent literature suggesting the analysis of urine odor for diagnostic purposes. This investigation aims to evaluate the possibility to compare the results of studies based on different approaches to be able in the future to identify those compounds responsible for urine odor alteration.

## 1. Introduction

Cancer is one of the major causes of mortality worldwide [1]. The International Agency for Research on Cancer (IARC), focusing on geographic variability across 20 world regions, estimated 18.1 million new cancer cases and 9.6 million cancer deaths in 2018 [1].

This large burden is projected to increase significantly in the near future with predicted 22 million new cancer cases and 13 million cancer-related deaths occurring annually by 2030 [2]. This increasing magnitude of cancer is a consequence of population growth and aging, but societal, economic, and lifestyle changes—linked to increasing human development—are likely to additionally increase the scale and alter cancer trends in the next decades [2].

Given low accuracies of current diagnostic procedures of some cancer types [3], researchers are rising to the challenge of developing innovative diagnostic tools able of identifying cancer during its early stage, that is the most curable one [4]. Effective screening tests should be non-invasive, easily accessible, quickly quantifiable, reliable, and reproducible. They should have high sensitivity, high specificity, low financial burden on patients and the lowest possible risk level [4].

In recent years, the deep understanding of biological structures and processes has revolutionized several aspects of cancer research, offering new opportunities for early cancer diagnosis before symptoms appear, when the tumor is at early stages.

Cell bioplastic transformation processes result in metabolic alterations, which lead to peroxidation of membrane components and consequent release of volatile organic compounds (VOCs) in biological fluids [4,5]. Therefore, the characterization of biological fluids seems to be an important chance for the improvement of cancer diagnosis and, in recent years, the interest in biological fluids chemical characterization, aimed at the identification of novel biomarkers, has increased significantly [4].

A biomarker is defined as a biological molecule found in body fluids, which can be termed as measurable indicator of pathological or physiological process of a condition or disease [6]. Based on information they provide, biomarkers are classified as:

- Diagnostic: used for risk stratification and early cancer detection.
- Prognostic: provide an indication of the likely progression of the disease.
- Predictive: used for predicting treatment measures to be taken on a patient.

Several literary studies [4,5,7] demonstrated that VOC profiles reflect any metabolic changes in response to inflammation, necrosis, cancer development and degeneration or microbiota alteration and that various cancers are associated with specific VOC profiles [8,9,10].

In the scientific literature, several papers underlined the fundamental role of biological fluids (breath [11,12], skin [12], sweat [13], feces [14], blood [15], and urine [16,17]) as sources of useful information for diagnostic, prognostic and predictive purposes. Different analytical techniques, such as nuclear magnetic resonance spectroscopy (NMR), high-performance liquid chromatography (HPLC), gas chromatography linked to mass spectrometry (GC-MS), sensor analysis with the electronic nose (e-nose) [7,18] and even canine scent detection [19,20], have been proposed for their characterization.

Exhaled breath contains hundreds of VOCs that can be attributed to either exogenous or endogenous volatiles. Exogenous volatiles include compounds inhaled from the external environment, related to food oral ingestion or derived from smoking [21]. Endogenous volatiles consist of blood-borne compounds released to the environment through the lungs and/or compounds produced by symbiotic bacteria [21]. Nonetheless, breath samples can be collected in extremely simple, painless and non-invasive way [21].

Also VOCs emitted from the skin can be investigated for identifying alterations related to metabolic disorder, bacterial alterations or diseases that can induce changes in both the quality and quantity of skin VOCs [21]. Skin VOCs sampling is non-invasive and simple: Samples are easily obtained by wiping the subject’s skin with an organic solvent or using an absorbent solid-phase microextraction fiber (SPME). However, care must be taken during sample collection to avoid contamination from the ambient air or from cosmetics [21].

Semen directly provides much information about prostate diseases, but sampling is very difficult and its variability complicates the identification of specific biomarkers [4].

Blood directly reflects the internal environment of the body, including nutritional, metabolic, and immune status [21]. However, sampling is invasive and the masking effects of high-abundance compounds worsen the identification of low-abundance proteins, potentially related to metabolic alterations [4].

The investigation of fecal VOCs may be the best non-invasive way of diagnosing gastrointestinal diseases, because human fecal samples represent dietary end-products resulting from digestive and excretory processes and intestinal bacterial metabolism [21].

Urine has been investigated for centuries as a source of useful information for the assessment of different diseases. Indeed, urine is the carrier of blood wastes and it is rich in intermediate or end products of many metabolic pathways [21]. Thus, urine may provide information not only from kidney and urinary tracts, but also from distant organs via plasma obtained through glomerular filtration [4,22].

Compared to other biological fluids, urine has the advantages of being inexpensive, rich in metabolites, easy to handle, and available in large amounts, without requiring invasive treatments for collection. However, potential biomarkers are present at very low concentrations and urine is characterized by high variability among patients depending on gender, age, hormonal status, diet, or physical activity [4]. Therefore, the experimental protocols to be adopted must be standardized.

This paper aims to provide an up-to-date state-of-the-art summary about cancer biomarkers proposed in the scientific literature, limiting the investigation to papers searching the biomarkers in urine samples and not in other biological fluids. Lung (LC), breast (BC), prostate (PrC), colorectal (CrC), gastric (GC), hepatic (HC), bladder (BlC), pancreatic (PaC), renal (RC), and testicular (TC) cancers have been considered.

The literature search covered a timeframe of publication between 2000 and 2019 for colorectal, prostate, hepatic, lung, bladder, renal, breast, pancreatic, and gastric cancers, while for testicular cancer older papers have been considered, due to the small amount of papers about urinary testicular-specific biomarkers available in the scientific literature.

Recent years have seen an exponential growth of metabolomics and the amount of papers proposing the investigation of urine for diagnostic purposes is huge. For this reason, the aim of this review is to resume the information that can be found in the scientific literature regarding the correlation of different cancer forms with the presence of specific metabolites in human urine in a schematic and easily interpretable form. Data are presented in the form of tables, which highlight proposed urinary biomarkers associated to different cancer types and the main differences between urine samples from control subjects and cancer patients reported in research studies, in order to enhance paper readability for the readers who want to get a deeper knowledge of this field.

Section 2 is structured in subsections relevant for different cancer forms sorted according to the tumor incidence. Subsections, besides a brief description of cancer stats and current diagnostic procedures, report schematic tables, which, aiming to highlight the main aspects of the literary studies investigated, report information about the population involved, the experimental method in terms of sample preparation and analytical techniques, a list of proposed urinary markers and main results presented.

The investigation of possible metabolic pathways alterations related to cancers development, which are extensively discussed in the scientific literature [23,24,25], are out of the scope of this paper. On the other hand, the originality of this review is that it focuses on the deeper investigation of the correlation between urine odor alterations and cancer presence and explores the possibility of identifying those urine cancer metabolites that are potentially responsible for those alterations through the combination of different approaches for urine characterization.

This type of investigation was encouraged by the promising results that have been recently achieved through innovative diagnostic tools based on the characterization of urine odor. Indeed, in recent years, many researchers proved that the urine odor analysis by means of the use of trained dogs or electronic noses allows the achievement of very high diagnostic accuracies, in some cases significantly higher than those of current diagnostic tests [17,26].

The last section proposes an extensive discussion about the main aspects of different studies, highlighting the results achieved and their criticalities. Moreover, the last section investigates cancer-related metabolites reported for different cancer forms by diverse research groups, looking for their odor properties and evaluating their possible responsibility for odor alterations.

## 2. Cancer Biomarkers in Urine

### 2.1. Lung Cancer

Lung cancer (LC) is the most common diagnosed cancer (11.6% of the total cases) and the leading cause of cancer death (18.4% of the total cancer deaths) among men [1].

Lung cancer incidence is related to environmental and/or genetic factors. Smoking is the main risk factor for LC, being linked to approximately 90% of LC cases [1,27].

LC is classified in two types: Small cell lung cancer (SCLC) caused by smoke and non-small cell lung cancer (NSCLC), which is the most diffused one [28].

Diagnostic methods, currently involved for LC diagnosis, are chest x-ray, computed tomography (CT), positron emission tomography (PET), sputum cytology, and bronchoscopy [29]. They are time-consuming, expensive, and quite dangerous due to radiation exposure [30]. Moreover, they do not provide exhaustive diagnostic information, thus biopsy or lung resection must be carried out to confirm the diagnosis and define the treatment plan [28,31,32].

Carcinoembryonic antigen (CEA), squamous cell carcinoma (SCC) antigen and neuron specific enolase (NSE) are serum markers, commonly used to detect lung cancer, monitor its progression and disease recurrence. However, for localized tumors, their diagnostic capabilities are relatively poor. In fact, most LC cases are found at an advanced stage, reducing the number of effective treatments and increasing the cancer related mortality [27,29,31].

Early-stage disease may allow a curative surgery with an adequate tumor extirpation, but no screening process, adopted up to now, are capable to detect the disease at a stage which improves the overall survival [28]. Indeed, the last 30 years have seen little improvement in the overall five-year survival rate for LC, with only 15% of patients living for at least five years after their initial diagnosis [27].

In recent years, researchers have proposed the combination of imaging tests with metabolomics in order to improve the specificity of the actual procedure [27]. Therefore, biological fluids have been investigated with the aim of identifying metabolites capable of providing information related to lung cancer presence and tumor development.

Table 1 summarizes literary papers investigating urinary biomarkers specific for lung cancer, focusing on the main aspects of the studies presented (i.e., population involved, experimental methods, and results).

### 2.2. Breast Cancer

Breast cancer (BC) is the second most common cancer form and the second leading cause of cancer related death in women [36]. In the Global Cancer Statistics for 2018 [1], about 2,088,849 BC new cases, mainly invasive tumors (i.e., about the 80%), and about 626,679 related deaths were estimated worldwide.

Breast cancer can start from different parts of the breast. However, most breast cancers start developing in the ducts that carry milk to the nipple (i.e., ductal cancer) or in the glands where breast milk is formed (i.e., lobular cancer). Although many types of breast cancer can cause lumps in the breast, most breast lumps may be related to non-cancerous breast tumors, which do not spread outside of the breast and are not life threatening [36].

The first step of current diagnostic procedure involves breast exam and imaging tests (mammogram, breast ultrasound, breast magnetic resonance MRI scan) to identify any lumps or abnormalities [37,38].

Since several researchers proved the higher efficacy of cancer treatments if breast cancer is detected at an early stage [39,40], women over 40 years old are recommended to undergo mammograms every year with the aim of detecting cancers before physical symptoms appear and treatments alternative to mastectomy and chemotherapy might be adopted.

Despite substantial increases in the number of cases of early-stage BC detected, screening mammography has only marginally reduced the rate of detection of advanced cancers. The imbalance suggests that there is substantial overdiagnosis, accounting for nearly a third of all newly diagnosed breast cancers, and that screening is having, at best, only a small effect on the rate of death from breast cancer [41].

In case of positive results of screening tests, the breast biopsy is carried out to confirm the cancer presence through the direct examination of the cell cytomorphology [42]. The choice of the type of the biopsy (i.e., core needle biopsy, fine needle aspiration (FNA) biopsy, surgical/large core biopsy, vacuum-assisted biopsy) to be performed depends on several factors: how suspicious the abnormality appears, the size, the shape and the location of the abnormality, the number of abnormalities present and the patient’s medical history.

Even with its high accuracy, breast biopsy is an invasive test, which carries some risks for patients and, since the consequential risk increases as the procedure becomes more invasive, core needle biopsy and fine needle aspiration (FNA) biopsy are preferred respect to surgical/large core biopsy and vacuum-assisted biopsy [43].

In recent years, many research groups started to investigate alternative methods to biopsy with the aim to develop non-invasive diagnostic tools [44,45,46,47,48,49]. Therefore, they started looking for novel BC biomarkers capable to improve current diagnostic procedure in biological fluids.

Table 2 summarizes the main aspects of recent literary studies proposing the investigation of urine samples aimed at the discovery of specific BC biomarkers.

### 2.3. Prostate Cancer

Prostate cancer (PrC) represents the most common cancer in men globally [50] and the fifth most frequent cancer in the world. In the Global Cancer Statistics for 2018 [1], about 1,276,106 PrC new cases, and about 358,989 related deaths were estimated worldwide.

PrC is an asymptomatic and slow growing tumor, and, thus, it is a perfect candidate for screening programs, whose purpose is the detection of cancer during its early stage, when the tumor is localized in the prostate and clinical treatments result more effective [4].

The current screening procedure for PrC detection involves the measurement of Prostate-Specific Antigen (PSA) serum level, the digital rectal examination (DRE), and the prostate biopsy.

The Serum Prostate-Specific Antigen (PSA) is currently the most important biomarker for the detection, follow-up, and therapeutic monitoring of prostate cancer. Although its use has been associated with a significant reduction in prostate cancer mortality, it has also resulted in the over-diagnosis and overtreatment of indolent prostate cancer [51]. Indeed, PSA test is characterized by a poor diagnostic accuracy (i.e., 30%) in terms of specificity and by a high false positive rate [52]: Many positive results are related to urinary tract infections or benign prostatic hyperplasia and not to prostate cancer. Additionally, serum PSA levels are affected by biologic variability that may be related to differences in androgen levels or prostate manipulation and may have distinct racial variation [53].

Therefore, patients with increased PSA values undergo prostate biopsy to confirm the cancer presence. The biopsy is an invasive test, which consists in the analysis under a microscope of tissue samples taken from the suspicious mass by surgery. It may carry out significant risks for patients and longer-term health issues such as bleeding, inflammatory, or infectious complications [54,55,56]. It also entails a low level of accuracy (i.e., only 30% detection rate at the first biopsy).

In addition to its invasiveness, the actual diagnostic procedure does not allow detecting the tumor before symptoms appear and when it is localized in the prostate. This reduces patients’ chances of surviving and increases patients’ management costs (e.g., need for extensive treatments and frequent hospital admissions) [4]. There is, thus, a need for more reliable, non-invasive method to diagnose prostate cancer.

Many studies regarding the investigation of novel PrC biomarkers have been published in the scientific literature. Table 3 summarizes literary works, focusing on the population considered, the experimental method involved, and the biomarkers proposed.

The schematization of the main aspects of literary studies highlighted that only partial results have been obtained until now, because, although different papers proposed the same metabolites as potential urine markers, results relevant for the quantitative urine characterization are discordant.

Sarcosine is the most debated biomarker among the ones proposed. Indeed, many authors [57,58] reported that its level in urine from PrC patients is higher than in control samples and that its classification performance is good, whereas other researchers [59,60] showed that changes in its concentrations between PrC and healthy men were not statistically significant.

### 2.4. Colorectal Cancer

Colorectal cancer (CrC) is the second most commonly diagnosed cancer in females and the third in males worldwide [71].

CrC incidence varies worldwide and is higher in more developed countries including Australia, New Zealand, Western Europe, and North America, revealing a strong contribution of environmental factors in disease development [71]. However, CrC incidence is increasing also in several Asian regions and in Eastern Europe, probably as a result of increases in smoking, adoption of unhealthy dietary habits and sedentary lifestyles [71]. CrC is thought to develop as a result of environmental factors and the accumulation of genetic and epigenetic alterations [72]. It occurs in inherited, familial and sporadic forms [72].

CrC related mortality is also high, with a global estimate of 600,000–700,000 deaths/year [71].

Current CrC screening tests can be broadly distinguished as early detection tools or cancer-prevention tools depending on their modes of action [6].

Detecting CrC at an early stage improves dramatically survival rates: five-year survival rate is 93% for stage I patients, but only 8% for stage IV patients [73].

Early detection tools involve fecal occult blood tests (FOBTs) and fecal immunochemical tests (FITs), which are non-invasive and cost effective [6]. However, the positive results are routinely recommended for endoscopy [6], which is the gold standard technique for CrC detection.

Nevertheless, its invasiveness, associated discomfort, potential risk of complications represent marked disadvantages [74] and lead many researchers to investigate and define novel methods based on specific biomarkers that would improve early CrC detection [75].

Although, in recent years, some CrC biomarkers, as carcinoembryonic antigen (CEA), have already been combined to current diagnostic tests, their sensitivity and specificity are relatively poor [76].

Table 4 summarizes the CrC biomarkers proposed in the scientific literature in the last 10 years, highlighting the analytical techniques adopted for their identification and quantification in urine samples.

### 2.5. Gastric Cancer

Stomach or gastric cancer (GC) was estimated to be responsible for over 1,000,000 new cases in 2018 worldwide and about 783,000 deaths, making it the fifth most frequently diagnosed cancer and the third leading cause of cancer death [1]. Rates are 2-fold higher in men than women. Several studied documented a strong environmental component in explaining the regional variation in stomach cancer incidence rates [1].

Helicobacter pylori is the main risk factor for stomach cancer, with almost 90% of new cases of noncardia gastric cancer attributed to this bacterium. However, also the dietary habits (i.e., low fruit intake, alcohol, and foods preserved by salting consumption, active tobacco smoking) engrave GC incidence risk [1].

GC can generally be distinguished into cardia and noncardia GC. Those two GC forms are characterized by different incidence rates: the number of noncardia GC new cases is decreasing thanks to prevention and the improvements in the preservation and storage of foods; whereas rates of cardia gastric cancer are increasing due to the rise of its risk factors, as obesity and gastroesophageal reflux disease (GERD) [1].

In general, since GC symptoms are non-cancer specific and may be related to other diseases as gastritis [78], GC is often diagnosed late, reducing the efficacy of treatments and patients’ chances of surviving [79]. However, for tumors detected when they are confined to the mucosal or submucosal layer, the 5 year survival rate is above 90% after surgical management [79]. This highlights the importance of appropriate screening in higher-risk population.

Currently, the standard diagnostic method for GC early detection involves gastroduodenal endoscopy (GE) as mass screening tool. GE adoption as mass screening tool has resulted in the reduction of GC-specific mortality and improved survival rates of GC patients [80]. However, data on the impact of GE screening programs on gastric cancer mortality are limited [81] and GE is an invasive and expensive tool.

Thus, the interest in the development of non-invasive and reliable tests capable of detecting GC in asymptomatic patients has increased significantly in recent years [78,79,82].

Many serum-and tissue-based biomarkers specific for GC have been identified through genomic and proteomic techniques, but only serum carcinoembryonic antigen (CEA) and carbohydrate antigen 19-9 (CA 19-9) have been proved to being clinically useful. Nevertheless, their sensitivities are poor [79].

Alternatively, urine has been investigated as potential source of specific GC markers that may allow early diagnosis and the discrimination between GC and benign gastric disease (BN). Table 5 summarizes the main aspects of research studies, investigating urinary metabolites related to stomach cancer, reported in the scientific literature.

### 2.6. Hepatic Cancer

In 2018, GLOBOCAN 2018 estimated that the liver or hepatic cancer (HC) would be the sixth most commonly diagnosed cancer and the fourth leading cause of cancer-related death worldwide, with about 841,000 new cases and 782,000 deaths annually [1].

Rates of both incidence and mortality are 2 to 3 times higher among men in most world regions; so liver cancer ranks sixth in terms of global cases and second in terms of deaths for males [1]. Liver cancer is diffused throughout the world, but in particular in Northern and Western Africa (Egypt, the Gambia, Guinea) and Eastern and South-Eastern Asia (Mongolia, Cambodia, Vietnam) [1].

Liver cancer incidence is increasing in Western Europe and in United States, maybe because of chronic alcohol use and chronic hepatitis C infection [84]. Diabetic and metabolite diseases of the liver have been known to contribute to this increasing incidence trend too.

Current methods for liver cancer diagnosis rely on imaging techniques (i.e., radiographic and ultrasound) that are not practical for mass screening tools aiming at early HC diagnosis [85], being costly and time-consuming [86].

Moreover, the current diagnostic procedure allows the detection of HC only at advanced stages. Consequently, treatments effectiveness is reduced, resulting in high mortality rate [87].

Because of the late diagnosis of liver cancers, the rate of postoperative recurrence at 5 years is significant (about 70%) [88].

Early detection of liver cancer and advances in surgical techniques might greatly improve short-term survival for those diagnosed with HC. Nevertheless, the identification of novel and more accurate biomarkers remains a very challenging task primarily due to the heterogeneity of disease development and progression [84].

Although alpha-fetoprotein (AFP), des-γ-carboxyrothrombin, the AFPL3 and the midkine (MDK) has been reported as specific HC biomarkers, researchers are still working on the identification of novel biomarkers because of low sensitivity and specificity of the actual procedures. Indeed, the AFP often leads to high rates of false positives and false negatives [89]: About 30% of primary liver cancer patients are AFP negative [87].

Novel biomarkers should be capable both to detect HC and monitor its progression, because methods currently available to investigate HC metastatic potential, such as cell adhesion, migration, invasion, and angiogenesis, are time-consuming, costly and unsuitable for clinical application [88]. Hence, a new rapid, cost-effective, and accurate prognostic method of invasion is needed.

Table 6 summarizes the main aspects of literary studies about urinary liver cancer biomarkers, highlighting population involved, techniques adopted and differences between healthy people and HC patients.

### 2.7. Bladder Cancer

Bladder cancer (BlC) is the seventh most common cancer and ninth leading cause of cancer-related death, with an estimated 549,000 new cases and 200,000 deaths in 2018 worldwide [1]. Bladder cancer is more common in men than women, with respective incidence and mortality rates of 9.6 and 3.2 per 100,000 in men: about 4 times those of women globally [1]. Incidence rates in both sexes are highest in Southern Europe (Greece, Spain, Italy), Western Europe (Belgium and the Netherlands), and Northern America. Considering only women, the highest rates are estimated in Lebanon.

At the time of diagnosis, about 70–80% of BlC are non-muscle-invasive bladder cancers (NMIBC), while the remaining 20–30% are muscle-invasive bladder cancers (MIBC) [92].

Although NMIBC and MIBC both originate from the urothelium in the urinary bladder, they have distinct clinical characteristics [92]. NMIBC is associated with good survival compared to other malignancies, although 30–50% of patients with NMIBC will eventually experience recurrence after transurethral resection (TUR) of the primary tumor, and 10–20% will progress to muscle-invasive bladder cancer (MIBC) [2]. In the case of MIBC, instead, patients often have poor outcomes despite systemic treatments, although radical cystectomy, radiation therapy, and chemotherapy are considered to be effective therapies [92].

Therefore, early diagnosis of BlC is essential to properly manage BlC and improve the efficacy of treatments and patients’ chances of surviving [92].

The current standard procedure for BlC detection and monitoring tumor progression and recurrence involves urine cytology, cystoscopy, and biopsy [92,93].

Urine cytology allows the detection of cancer or pre-cancer cells through a microscope screening of urine samples. However, its low sensitivity towards low-grade tumors reduces test reliability, although it is a perfect tool for the detection of high-grade bladder cancers [92,93,94].

Cystoscopy, i.e., the endoscopy of bladder via urethra, is an expensive, invasive and painful test, which may easily miss high-grade tumors [92,93]. Indeed, carcinoma in situ (CIS) looks like red mucosal spots typical of inflammatory lesions and may be confused [94]. Therefore, if an abnormal area is seen during cystoscopy, the patient undergoes biopsy to confirm the diagnosis. Although recent advances in biopsy technology, this exam is not a perfect tool and it may miss especially small tumors.

Thus, new diagnostic approaches that will improve the diagnostic accuracy of current procedure and the discrimination between non-malignant conditions and MIBC from NMIBC are needed [92]. In recent years, many efforts have been made to develop a non-invasive and less expensive tool for diagnosis, prognosis and monitoring of the treatment efficacy [93].

For this purpose, different analytical techniques, such as LC-MS, GC-MS, and NMR, have been proposed in the scientific literature for the chemical characterization of urine aimed at the identification of potential markers [92].

Table 7 proposes a schematization of literary studies investigating potential urinary BlC biomarkers, highlighting the main aspects of research studies presented in the literature.

### 2.8. Pancreatic Cancer

Pancreatic cancer (PaC) is the eighth leading cause of cancer death in both males and females, with 432,000 deaths and 459,000 cases worldwide [1]. Incidence rates are higher in countries with high human development index (HDI), mainly in Europe, North America, and Australia/New Zealand [1].

The risk of developing pancreatic cancer goes up as people age: about 80% of PaC patients are at least 60 years old and 71 is the average age at the time of diagnosis [97].

The most frequent pancreatic malignant tumor is the pancreatic ductal adenocarcinoma, representing the 85% of all reported cases [98], which could be associated to nodal metastasis and hepatic, bone or pulmonary metastasis [97]. PaC risk may be related to genetics factors (family history) and/or environmental factors, as smoking, alcohol consumption, chronic pancreatitis, obesity, and diabetes [97,99].

Rarely pancreatic cancer manifests specific symptoms when the tumor is at an early stage [100], thereby resulting in late diagnosis. Indeed, when symptoms like asthenia, jaundice, abdominal pain and weight loss, appear, patients already have an advanced pancreatic neoplasia [97].

Surgery is still the only curative therapy for pancreatic cancer [101]. However, although successful surgery of the pancreas is possible, only the 20–25% of patients are diagnosed at early disease stages when resection is effective [102]. Moreover, even in case of successful surgery, the median survival after surgery is only 17–23 months, due both to the resistance of pancreatic cancer to chemotherapy [98] and to the advanced stage diagnosis [102].

Current PaC diagnostic techniques are imagine techniques as computed tomography (CT), positron emission tomography-CT, magnetic resonance imaging (MRI), endoscopic retrograde cholangiopancreatography (ERCP), and endoscopy ultrasonography (EUS), which is sometimes associated to biopsy used for grading tumor histology [98,101,103]. However, these techniques are not so effective in detecting tumor of less than 2 cm in diameter [102].

The measurement of the Carbohydrate antigen 19-9 (CA 19-9) serum level is commonly used as a complementary test in the current diagnostic protocol [101]. However, although its sensitivity can reach 80%, the CA 19-9 has low specificity and it is unsuitable for the detection of PC at resectable stages [102].

Also CEA, CA242, MIC-1, M2-PK, ADAM9, PBF-4, PNA-binding glycoprotein, MMP-2, ICAM-1, CEACAM1 have been identified as candidate biomarkers [102,103,104]. However, their adoption is associate to high false negative rates [100].

In the scientific literature, metabolomics, genomics and post-genomic technologies have been proposed for the identification of potential PaC markers in biological fluids with the aim to define novel non-invasive and accurate diagnostic tool to enhance the early PaC detection [100]. Many authors proved the possibility to discriminate between healthy and PaC subjects through the investigation of blood, tissue and saliva [105,106,107,108,109,110,111,112,113,114] by means of NMR, GC/MS, HPLC/MS, UPLC/MS and CE. However, these studies involved relatively small populations and considered a small number of early stage or resectable cancers [102]. Other authors proposed the characterization of bile and pancreatic fluids, but their handling requires costly and invasive techniques [104].

Only few research groups proposed the investigation of urine samples and proposed specific PaC urinary biomarkers (Table 8), whereas Arasaradnam et al. [115], proposed the adoption of the ion mobility spectrometry for the characterization of the urine volatiles, achieving a sensitivity of 91%, a specificity of 83% and an accuracy of 92%.

### 2.9. Renal Cancer

The renal or kidney cancer (RC) is one of the ten most common cancers in both men and women, although it is more frequent in men than in women [1]. For 2019, the American Cancer Society estimated about 73,820 new cases of kidney cancer and about 14,770 deaths related to this form of tumor [121].

Actual diagnostic procedure involves urinalysis and blood tests to look for blood traces and measure the levels of calcium and liver enzymes, which might be altered due to kidney cancer presence.

Urine cytology is carried out on urine samples to identify cancer cells eventually present. In case of abnormal results, patients undergo imaging tests, capable to provide useful information about tumor size, shape, and location, producing detailed cross-sectional images of suspected areas.

Computed tomography (CT) scan is the most common imaging tool involved for renal cancer diagnosis, but also magnetic resonance imaging (MRI) scan, ultrasound, positron emission tomography (PET) scan, intravenous pyelogram or angiography can be used [122,123].

Unlike other cancer types, biopsy is not often used for kidney cancer diagnosis, since imaging tests provide enough information to evaluate the need of surgery. However, in case of small tumors, biopsy is carried out to evaluate alternative treatments to surgery [122].

Since the RC is an asymptomatic tumor at early stages, in general current diagnostic procedure allows detection when symptoms appear and prognosis is poor [124]. Therefore, the identification of a screening biomarker has the potential for substantial health benefit.

In recent years, many researchers have proposed urine chemical characterization for metabolic profiling and identification of specific RC biomarkers. Literary works reported in Table 9 proposed novel urinary biomarkers specific for RC. Some authors [124,125] proposed an innovative approach, without attempting to identify all detected peaks, but rather than focusing on evaluation of the use of mass spectrometric and peak processing techniques for the development of innovative diagnostic tests for RC. Their approach was based on the idea that a large group of potential biomarkers was more likely to evolve patterns for disease recognition. The decisional models proposed by both research groups [124,125] achieved diagnostic accuracies above 88%.

### 2.10. Testicular Cancer

The Global Cancer Statistics 2018 [1] estimated for testicular cancer (TC) 71,105 new cases and 9507 related deaths. TC is more common cancer in young adults and potential risk factors include undescended testis (cryptorchidism), personal or family history of testicular cancer, age, ethnicity, and infertility [129].

About 1% of testicular cancers are neuroendocrine tumors, commonly known as carcinoid tumors, which are mostly primary tumors of the testes and rarely are metastasis to the testes from other organs. As opposed to common testicular cancer, carcinoid tumors can affect men of all ages [130].

As first steps of the current diagnostic procedure for testicular cancer, the patient undergoes a physical exam to check testicles for lumps, swelling, hardening, or tenderness and identify abnormal masses and his history and health habits are examined [131].

In case of abnormal results of the physical exams, an ultrasound exam of the testes and blood tests for specific-tumor markers are performed to evaluate if abnormalities are related to benign conditions (i.e., hydrocele or varicocele) or to testicular cancer [131].

Alpha-fetoprotein (AFP), human chorionic gonadotropin (HCG) and lactate dehydrogenase (LDH) are used as serum markers of testicular cancers. Indeed, rises in levels of AFP and HCG may allow the detection of the tumor and the identification of its type: non-seminomas or pure seminomas; while high LDH levels indicate a widespread disease [132].

Biopsy is rarely done for diagnosing testicular cancer because of the high risk of spreading cancers. Thus, based on ultrasound and blood marker tests, the doctor will very likely recommend inguinal orchiectomy, which consists in the removal of the entire testicle through an incision on the groin [131].

Although the scientific literature investigating testicular cancer urinary biomarkers is not so huge, this kind of tumor is reported for completeness of this review paper. Table 10 reports the main aspects of the scientific papers.

## 3. Discussion

### 3.1. Design of the Experiment

In the scientific literature, it is commonly known that cancers are caused by uncontrolled growth of abnormal or mutated cells. Mutated cells contain broken genetic information, which make them resistant to apoptosis and capable of undergoing faster replication [135]. Due to genetic alterations, the pathways through which cancer cells acquire and replenish their metabolic needs are different from those of normal cells. This results in qualitative and quantitative alterations of the metabolomics profile of urine samples from healthy subjects and cancer patients, which can be investigated for early cancer diagnosis.

Therefore, with the purpose to identify specific metabolites relevant for those metabolic alterations, most of the scientific papers about novel biomarkers investigation propose the comparative analysis of urine samples from healthy subjects and cancer patients and, in general, the population involved is divided into control and sick groups.

In many cases, the control group includes only healthy subjects with no cancer-related pathologies. However, some authors [47,61,63,67,68,78,84,90,91,92,125,126,136] investigated also the specificity of proposed novel biomarkers towards the cancer type of interest, including in the study also other tumor types or interfering diseases (such as benign prostatic hypertrophy BHP, stones, cysts, oncocytoma, adenoma).

In almost all papers, the cancer patients were divided into subclasses according to their cancer stage with the aim to identify urinary specific-cancer metabolites useful for both tumor detection and prognosis, since one of the main issues related to current screening tools is the over-diagnosis of patients [137,138,139,140]. Indeed, due to their low specificity, actual diagnostic tests may provide positive results also for non-cancerous diseases and do not allow the discrimination between aggressive and indolent cancers, especially for prostate, bladder, liver cancers. This entails the overtreatment of patients affected by non-life-threatening tumors and consequently the increase of patients’ management costs (e.g., hospitalization, frequent control tests) [4].

Some authors [62,84] studied also the disease recurrence, analyzing urine samples from patients who developed early biochemical recurrence within two years of surgery and those who remained recurrence-free after more than five years. These researches had the aim to identify specific compounds capable of predicting tumor recurrence with high diagnostic accuracy and thus guide the post-surgery therapeutic plan.

This review focuses also on the experimental protocols suggested in the scientific literature for urine sample preparation and its chemical characterization.

Diverse analytical techniques, involving the analysis of liquid urine or its gaseous headspace, were proposed. The most common ones were the gas or liquid chromatography combined to mass spectroscopy (GC-MS and LC-MS), but also other techniques, as IELC, IMS, or NMR, allowed to achieve very promising results in terms of diagnostic sensitivity and specificity.

In almost all papers, the experimental method involved the freezing and the pre-treatment of urine samples before analysis. The freezing allowed to prevent the alteration of urine composition during the storage due to bacterial activity, while pre-treatments were carried out on liquid urine samples to optimize the following characterization of urine composition.

The type of pre-treatments strictly depends on the analytical technique involved. In general, the pre-treatments consist in the centrifugation of liquid urine just after the collection or after thawing and the addition of chemicals (e.g., NaOH, HCl) to maximize the headspace enrichment in case of GC-MS or facilitate the detection of target compounds (supposed to be indicators of cancerous metabolic alterations) in case of liquid urine analysis.

In the scientific literature, both the qualitative and quantitative characterizations of urine composition were proposed. Some authors [63,66,69,70,78,124,127,141] suggested also the adoption of data processing techniques of the multivariate statistics, as ANOVA, PLS-DA, LDA, for the elaboration of analytical results to investigate the urine metabolic fingerprint of different cancer types with the aim of identify specific metabolites capable of both diagnosing tumors and monitoring their progression.

The critical investigation of the scientific literature in this field highlighted that the chemical characterization of urine composition has the potential to provide various cancer biomarkers, which could contribute to the development of new tests to allow early detection and avoid invasive diagnostic procedure, thereby reducing the economic burden of unnecessary or ineffective treatments.

Unfortunately, since this research field is new and continuously moving, results published until now in the scientific literature are not exhaustive and the discoveries of literary researches cannot yet be applied in everyday clinical practice. Indeed, results need to be validated, and method uncertainty evaluated.

Most existing studies have been compromised due to small number of samples analyzed and the lack of well-defined control groups [142]. Indeed, to achieve the ultimate goal of practicality for clinical applications, the relationship between the presence in urine of specific metabolites and the presence of cancer needs to be validated for large sample sizes with appropriate control groups. For this purpose, the experts in the field strongly recommend the inclusion in the control group of patients with other diseases or disorders with clinical and metabolic profiles close to those of the cancer of interest, in order to assess the specificity of the potential new diagnostic tool towards the cancer of interest [142].

Moreover, in the validation phase, the results’ transferability between different experimental protocols should be assessed with the aim to resolve disparities among the findings pointed out by different studies investigating the same disease.

### 3.2. Recurrent Cancer Biomarkers and Their Levels in Urine

Although the critical aspects above mentioned, the in-depth analysis of literary works about urinary cancer biomarkers highlighted that some metabolites were proposed by different authors as qualitative and, in some cases, quantitative indicators of the presence of different cancer types.

Table 11 reports a schematization of recurrent urinary cancer biomarkers proposed in the investigated literature and information regarding their concentration trends in urine samples from cancer patients with respect to controls. As already mentioned, it is important to highlight that this research area is continuously moving and evolving, so the data here reported are susceptible of variations with future discoveries in this field. Thus, current data shall be considered as uncertain, although precise data about their uncertainty range are not yet available.

Glycine, serine, threonine, alanine, and phenylalanine have been proposed as biomarkers related to colon [77], prostate [64,65,70,143], liver [84,89,90], breast [48], bladder [93], lung [33], and gastric [83] cancers. For all cancer types investigated, authors reported that threonine and glycine levels were higher in cancer patients than in healthy subjects, whereas no unique trend has been proposed for serine and alanine. Phenylalanine concentration was higher in cancer patients than controls for all tumors considered, except for prostate cancer, for which the concentration trend in cancer patients with respect to controls was the opposite.

Other recurrent biomarkers proposed in scientific papers investigating prostate [65,70], liver [84,89,91], bladder [93], gastric [79], colon [73,76,77], lung [33], and breast [46] cancers were tyrosine, indole, hippurate, hydroxyhippurate, tryptophan, kynureate, lactate, lactic acid, indole acetate, indolectate, taurine, hypotaurine, and quinate. Tyrosine has been proposed as a biomarker capable to diagnose cancer (i.e., the tyrosine urine level in cancer patients was higher than in controls) and discriminate between high-risk patients and low-risk patients (i.e., the tyrosine urine level was higher in low-risk cancer patients). Instead, hippurate, taurine, and hydroxytryptophan levels in urine were higher in cancer patients’ urine with respect to controls, allowing the discrimination between controls and cancer patients.

Those recurrent biomarkers are involved in metabolic pathways associated with cells demand and production of energy, as glycolysis, Krebs cycle, oxidative phosphorylation (OXPHOS), glutamine metabolism, fatty acid oxidation, nucleic acid synthesis, lipid synthesis, and amino acid metabolism, which are altered in case of cancer presence. Indeed, many researchers proved that cancer modifies these metabolic pathways involved in the production of energy [143], to satisfy the uncontrolled growth of mutated cells, although mechanisms on which these phenomena are based on have not been fully understood yet.

Also citrate, isocitrate, putrescine, succinate, aconitate, citrulline, malate, oxaloacetate, valine, leucine, isoleucine, arginine, creatinine and its derivative, involved in the tricarboxylic acid cycle (TCA), have been proposed as biomarkers for prostate [57,58,61,62,64,65,67,68,70], bladder [92,93,94], breast [48], gastric [79], liver [84,89,90,91], renal [128], colon [73,76,77], and lung [33] cancer. However, different studies presented different results about their urinary concentration levels of most of those biomarkers, except for arginine and citrate levels, which have been reported to be higher and lower respectively in cancer patients with respect to controls.

Some biomarkers involved in the TCA cycle have been recognized also capable to stage cancers: isoleucine for gastric cancer, leucine for bladder cancer, cysteine for liver cancer, and valine for colon and gastric cancers.

Certain research papers about liver [84,89], breast [47], prostate [61], bladder [93], gastric [79], and colon cancers [77], highlighted also the correlation between urine levels of adenosine, uridine, cytidine, purine, xanthine, guanine, adenine, guanosine, and inosine and cancer presence, but no information about their concentration in urine was provided.

The other metabolites significantly differentiating cancer patients from control subjects were organic acids: aspartic, malic, succinic, xylonic, kynureic, octanedionic, butanedionic, heptanedionic, ethanedioic, propanoic, butanioc, trihydroxypentanoic, glicholic, uric, citric, nicotinic, hippuric, and acetic acid. Those biomarkers have been proposed for colon [77], prostate [60,61,65,70], liver [84,89,90], gastric [83], bladder [93,94,96], and lung [33] cancer. Even though many authors underlined the significance of those metabolites for cancer diagnosis, different concentration levels between healthy subjects and cancer patients have been reported in different studies investigating the same cancer forms.

In the scientific literature investigated, besides urinary metabolites related to many cancer forms, also specific biomarkers, such as sarcosine for prostate cancer [57,58,59,61,63,64,65], NGAL, MMP-9 and ADAM 12 for breast cancer [44,45], HCG for testicular cancer [133,134], h.KIM-1 for renal cancer [126] or Prostaglandin E2 for gastric cancer [82] have been reported.

### 3.3. Investigation of the Correlation between Urine Odour Alteration and Cancer Presence

In recent years, given the criticalities related to the chemical characterization of urine composition and the disparities among different literary works, many researchers started to investigate the possibility to adopt sensorial and sense-instrumental analysis for cancer diagnosis.

In particular, this innovative approach involves the analysis of the odor emanated from urine with the aim detect the presence of the tumors before symptoms appear. This type of analysis provides the characterization of odorous headspaces of urine samples as a whole, providing their “olfactory fingerprints” without identifying the chemical composition of the mixture [4].

This approach has the advantage of simplifying the challenging task of the urine characterization by means of cheaper and faster analytical techniques [4]. Moreover, some of these innovative methods achieved diagnostic sensitivity and specificity higher than the current diagnostic tools [4,16,144,145,146,147]. As an example, Taverna et al. published in 2015 [26] a pilot study concerning the adoption a rigorous procedure for training two German Shepherd Explosion Detection Dogs to identify a pool of VOCs specific of prostate cancer emanated from urine samples, thereby defining an innovative method for PrC diagnosis. The dogs were taught to sit in front of the cancerous sample after sniffing a set of six urine samples, including one PrC sample and five controls. Urine samples, stored at −20 °C, were defrosted for the analysis and housed in circular perforated metal containers, which were placed in thermally sealed plastic packets to avoid any contamination. Taverna’s research involved a huge and multi-faceted population (i.e., 902 participants), including also men and women suffering from different tumors. Diagnostic test performance was evaluated, considering the whole population, after excluding females and considering only control men older than 45 years. In all cases, sensitivity was higher than 98% and specificity was over 96%.

An example of research concerning urine odor analysis by e-nose for cancer diagnosis was published by Horstmann et al. in 2015 [148]. They evaluated the potential of an electronic nose system equipped with MOS sensors for the detection of bladder cancer. Fresh voided urine was collected from 15 patients with the clinical suspicion of primary or recurrent bladder cancer and 21 patients without bladder cancer but with benign urological diseases. The results of this pilot study revealed the high potential of the electronic nose in the detection of bladder cancer with an overall sensitivity of 75% and specificity of 86% necessitating further investigations [7].

This paper does not aim to provide further analysis of the scientific literature regarding the adoption of odor analysis for diagnosing cancer, since comprehensive review papers on this subject have been recently published [7,149]. Nonetheless, the examples here described are representative of the potential of urine odor analysis for diagnostic purposes. Indeed, those very promising results prove the existence of a correlation between urine odor properties and the presence of certain cancer forms. However, this correlation is not yet fully understood and the urine components responsible for these odor alterations have still not been identified.

This review, besides the intent to summarize literary results about novel cancer biomarkers, aims to deeper investigate this aspect. For this purpose, one original aspect of this paper is that it tries to correlate the odor properties of potential urinary biomarkers proposed in the scientific literature for different tumor forms.

The term “odor” refers to the sensation caused by the interaction of some chemical compounds of a gaseous mixture, commonly named “Odorants”, with the mammalian olfactory receptors (ISO 5492). The odorants are molecules smaller than 300 amu, characterized by high volatility, which allows them to reach the upper part of the nose and interact with the olfactory receptors.

An odorant is capable to provoke a stimulus in the olfactory system, when it reaches in the atmosphere the “Perception or Odor Threshold” (OT), which is defined as the minimum concentration of the odorant that is perceived by 50% of the exposed population.

In the scientific literature, the OT relevant for diverse odorous pure substances have been proposed [150,151]. However, the tabulated OT values for the same compound are not unique and may differ in order of magnitude, probably due to the different methods adopted for their determination. Nevertheless, the tabulated OT can be considered for a preliminary screening of the odor properties of urine metabolites for which a correlation with various cancer forms has been proved.

This review proposes the investigation of the odor threshold and qualitative description of recurrent biomarkers, fulfilling odorants characteristics (e.g., volatility) with the aim to explore the possibility of identifying those metabolites that tend to alter urine odors, and which may therefore be detectable by trained dogs or electronic noses.

Tabulated OT values refer to human olfaction, and are therefore not directly explicative of the high accuracies in the detection of different types of tumors achieved by trained dogs [152]. Indeed, dogs’ olfactory system, which is significantly more powerful than human one, is capable of detecting odor thresholds as low as part per trillion, thanks to the huge dimension of their olfactory epithelium (up to 170 cm^2^ vs. 10 cm^2^ in humans), the huge number of olfactory receptors (over 200 million vs. nearly 5 million in humans) and the dense innervations of their olfactory mucosa [153].

Table 12 reports the list of cancer biomarkers considered, their odor thresholds and qualitative descriptions and the cancer type for which the correlation has been proven.

Many recurrent biomarkers have characteristic odors, described as unpleasant, pungent or nauseating, and, in some cases (e.g., acetic acid, amine derivatives, pyridine, cresol), their OT are very low. Thus, cancer urinary metabolites can be detected and recognized by the human olfaction at very low concentrations (i.e., ppb level).

These odor properties might confirm the results achieved in recent research studies involving the urine odor analysis by mean of trained dogs or electronic noses to discriminate between control subjects and cancer patients. However, this research field is still growing and, given the disparities among different literary studies about biomarkers concentrations in urine from cancer patients and controls, in this phase no specific considerations aimed at the identification of those molecules detected by trained dogs or electronic nose systems can be made.

Nevertheless, these observations prove the possibility to combine the traditional approach based on the chemical characterization of urine composition with the urine odor analysis, with the purpose of adding useful information to the challenging task of cancer biomarker identification. This may provide an innovative pathway for the development of new and more accurate cancer diagnostic tools.

Therefore, despite the difficulties associated with the development of innovative and reliable diagnostic techniques, a significant increase of the research in this field—and hopefully the successful introduction of some of these techniques in clinical diagnosis—in the near future is to be expected due to the high social and economic impact that new technologies for early diagnosis of cancer might have in today’s culture [4].

## 4. Conclusions

The critical analysis of the scientific literature about urinary cancer biomarkers highlighted the potentialities of metabolomics for the development of innovative cancer diagnostic tool, capable to detect the diseases at early stages and improve the diagnostic accuracy of current procedures. However, results achieved until now are not exhaustive and need to be validated for large sample sizes with appropriate control groups, including also other diseases or disorders with clinical and metabolic profiles close to those of the cancer of interest.

Given the very promising diagnostic accuracies published in recent studies, involving an innovative approach based on urine odor analysis for the development of new diagnostic tool, this paper aimed to deeper investigate the correlation between urine odor alteration and cancer presence. Thus, the investigation of the odor properties of urine biomarkers, for which a correlation with different cancer forms has been discovered, are here reported.

This analysis pointed out that some recurrent urinary metabolites can be detected and recognized by the human olfaction at very low concentrations, suggesting the possibility to combine the traditional approach based on the chemical characterization of urine composition with the urine odor analysis with the purpose of simplifying the challenging task of cancer biomarker identification.

## Figures and Tables

**Table 1:** Literary studies investigating urinary biomarkers for lung cancer. GC-MS: gas chromatography linked to mass spectrometry; SPME: solid-phase microextraction fiber; LC: lung cancer.

Authors (Year) [Ref]	Population	Experimental Method	Biomarkers	Results
Yang et al. (2010) [33]	35 LC patients (47–75 years) 32 controls (34–67 years)	Sample preparation: thawing, centrifugation, treatments with chemicals and film filtration Analytical technique; LC-MS	Taurine Hippuric acid Pipecolic acid Alpha-M-phenylacetyl-L-glutamine Valine Proline betaine isotope Phenylalanine Betaine Carnitine Leucylproline 3-Hexaprenyl-4-hydroxy-5-methoxybenzoic acid	All of these biomarkers are up-regulated in LC patients than controls
Guadagni et al. (2011) [34]	10 LC patients 25 controls	Sample preparation: thawing, treatments with chemicals and extraction by SPME Analytical technique: GC-MS	Hexanal Heptanal	Hexanal concentration is higher in LC patients than in controls, while heptanal concentration is not so different between LC patients and controls
Hanai et al. (2012) [35]	20 LC patients (59–77 years) at different stages 20 controls: (38–62 years)	Sample preparation: thawing, centrifugation, filtration and extraction by SPME Analytical technique: GC-TOF MS	Tetrahydrofuran 2-chloroethanol 2-pentanone 2-methylpyrazine Cyclohexanone 2-ethyl-1-hexanol 2-phenyl-2-propanol Isophorone	All of these biomarkers are up-regulated in LC patients than controls, the most significant are six of them. 2-pentanone is important for the differentiation between patients with adenocarcinoma and those with squameous cell, because in the first one 2-pentanone is higher.

**Table 2:** Literary studies investigating urinary biomarkers for breast cancer.

Authors (Year) [Ref]	Population	Experimental Method	Biomarkers Proposed	Results
Fernandez et al. (2005) [44]	22 breast cancer (BC) patients 27 controls	Sample preparation: Thawing, pre-treatment with chemicals and centrifugation Analytical technique: Gelatine zymography	Neutrophil Gelatinase-Associated Lipocalin (NGAL) Matrix metalloproteinase (MMP-9)	Increased levels of NPAL and MMP-9 were found in urine from women with breast cancer, resulting in stimulation of tumor growth
Pories et al. (2008) [45]	148 BC patients at different stages 80 controls	Sample preparation: Thawing, pre-treatment with chemicals and centrifugation Analytical technique: Zymography Immunoblotting ADAM 12	Matrix metalloproteinase (MMP-9) ADAM 12	ADAM 12 and MMP-9 are highly significant predictors of breast cancer, which can be used in conjunction with the Gail model, allowing the discrimination of control women from patients affected by breast cancer with sensitivity and specificity above 97%.
Nam et al. (2009) [46]	50 BC patients at different stages 50 controls	Sample preparation: Pre-treatment with chemicals and extraction Analytical technique: AMDIS	Homovanillate; 4-hydroxyphenylacetate; 5-hydroxyindoleacetate; urea	Homovanillate, 4-hydroxyphenylacetate, 5-hydroxyindoleacetate and urea were identified to be different in cancer and control urine
Woo et al. (2009) [47]	10 BC patients 12 cervical cancer 9 ovarian cancer 22 controls	Sample preparation: Thawing and pre-treatment with chemicals Analytical technique: GC-MS LC-MS	8-hydroxy-2-deoxyguanosine 5-hydroxymethyl-2-deoxyuridine	Urinary biomarkers were found by metabolite profiling and validated by multivariate data analysis and ANOVA. 8-hydroxy-2-deoxyguanosine and 5-hydroxymethyl-2-deoxyuridine levels were increased probably as consequence of oxidative DNA damage involved in cancer development.
Slupsky et al. (2010) [48]	48 BC patients (30–86 years) at different stages 72 controls (19–83 years)	Sample preparation: Thawing and pre-treatment with chemicals Analytical technique: h-NMR	Creatinine; Acetate; Succinate; Isoleucine; Sucrose; Leucine; Urea; Ethanolamine; Dimethylamine; Creatinine; Alalnine; Uracil; Valine	Urinary metabolites levels were decreased in breast cancer patients with respect to controls.
Silva et al. (2012) [49]	26 BC patients 21 controls	Sample preparation: Thawing and pre-treatment with chemicals Analytical technique: GC-qMS	4-carene; 3-heptanone; 1,2,4-trimethylbenzene; 2-methoxythiophene Phenol	4-carene, 3-heptanone, 1,2,4-trimethylbenzene, 2-methoxythiophene, Phenol levels in urine samples from breast cancer patients with respect to controls.

**Table 3:** Literary studies investigating urinary prostate cancer biomarkers.

Authors (Year) [Ref]	Population	Experimental Method	Biomarkers	Results
Sreekumar et al. (2009) [57]	59 prostate cancer (PrC) patients 51 controls	Sample preparation: organic and aqueous extractions of liquid urine, drying on TurboVapR Analytical techniques: LC-MS GC-MS ID GC-MS	Sarcosine Uracil; Kynurenine Glycerol-3-phosphate Leucine Proline	Sarcosine was significantly higher in urine sediments (AUC 71%) and supernatants (AUC 67%) of PrC patients; Uracil, Kynurenine, Glycerol-3-phosphate, Leucine, Proline were elevated upon disease progression.
Jentzmik et al. (2010) [59]	107 PrC patients at different stages 45 controls	Sample preparation: Centrifugation, no info about headspace enrichment Analytical techniques: Ez:faast amino acid analysis (SPME followed by GC-MS)	Sarcosine	Median Sarcosine/creatinine was 13% lower in PrC patients than in controls
Jiang et al. (2010) [58]	5 PrC patients 5 controls (18–78 years) without kidney disease	Sample preparation: thawing of frozen samples and pre-treatments with chemicals Analytical techniques: HPLC/MS/MS	Sarcosine Proline Kynurenine Uracil Glycerol-3-phosphate Creatinine	The ratio nM _metabolites_/µM _creatinine_ was higher in urine from PrC patients with respect to controls
Wu et al. (2010) [61]	20 PrC patients at different stages 28 controls: 8 BHP 20 healthy male	Sample preparation: thawing of frozen sample, centrifugation and treatments with chemicals and membrane filtration Analytical techniques: ID GC-MS	Sarcosine; Propenoic acid Pyrimidine Dihyroxybutanoic acid Creatinine Purine Glucopyranoside Ribofuranoside Xylonic acid Xylopyranose	PrC patients average sarcosine value were 13% higher than healthy controls and 19% higher than BPH controls. Also propenoic acid, dihyroxybutanoic acid, creatinine, and xylonic acid, dihyroxybutanoic acid and xylonic acid, concentrations were higher in PrC patients.
Stabler et al. (2011) [62]	54 PrC patients: 29 recurrent free 25 PrC recurrence	Sample preparation: no info Analytical techniques: GC-MS	Cysteine Homocysteine Dimethylglycine Sarcosine	Higher serum homocysteine, cystathionine, and cysteine levels independently predicted risk of early biochemical recurrence and PrC aggressiveness. The methionine further supplemented known clinical variables to increase sensitivity and specificity.
Bianchi et al. (2011) [63]	33 PrC patients (clinically localized PrC) 23 controls: 13 healthy 10 BHP	Sample preparation: no info Analytical techniques: SPME/GC-MS	Sarcosine N-ethylglycine	µg _Sarcosine_/g _Creatinine_ discriminates between healthy, BHP and PrC patients. The model built considering a cut-off 179µg/g achieved a sensitivity of 79% and a specificity of 87%.
Shamsipur et al. (2012) [64]	12 PrC patients 20 controls (30–65 years)	Sample preparation: thawing and centrifugation Analytical techniques: DDLLME/GC-MS	Sarcosine Alanine Proline Leucine	Sarcosine mean concentrations were higher in PrC patients; Leucine mean concentration was lower in PrC patients
Heger et al. (2014) [65]	32 controls 32 PrC patients at different stages	Sample preparation: pre-treatment with chemicals, centrifugation Analytical techniques: IELC IEMA	aspartic acid; threonine; methionine; isoleucine; leucine; tyrosine; arginine; sarcosine; proline; uric acid; urea; PSA; fPSA; glucose; creatinine; pH; total proteins; concentrations of K+, Na+, Cl−	All amino acids were increased in PrC patients, except for phenylalanine amounts. In controls, higher levels of K+ and uric acid and lower levels of urea and creatine were detected. PSA and free PSA were below the detection limit in controls.
Khalid et al. (2015) [66]	59 PrC patients (50–88 years) 43 controls (41–81 years)	Sample preparation: Thawing of frozen samples, pre-treatment with chemicals and incubation at 60 °C in a water bath for 30 min Analytical techniques: SPME/GC-MS	2,6-dimethyl-7-octen-2-ol Pentanal 3-octanone 2-octanone	Except for pentanal, all of these compounds were down-regulated and/or less frequently present in the urine samples from PrC patients. Model AUC based on 4 biomarkers discovered was 63–65%, while it was 74% (RF) and 65% (LDA) if combined with PSA level.
Tsoi et al. (2016) [67]	66 PrC patients at different stages of disease 99 controls: 88 BHP 11 healthy	Sample preparation: Thawing, centrifugation, pre-treatment with chemicals Analytical techniques: UPLC-MS/MS	Putrescine (Put) Spermidine (Spd) Spermine (Spm)	Normalized Spd was significantly lower in PrC than in BHP patients and controls The AUC for normalized Put, Spd and Spm were found to be 0.63 ± 0.05, 0.65 ± 0.05 and 0.83 ± 0.03 respectively
Sroka et al. (2016) [68]	25 PrC patients at different stages of disease 25 controls with BHP	Sample preparation: Pre-treatment with chemicals, centrifugation, incubation at 55 °C for 10 min. Analytical techniques: LC-ESI-QqQ-MS	Arginine Homoserine Proline Tyramine	In PrC samples, higher concentrations of arginine both before (P = 0,018) and after (P = 0,009) prostate massage and higher levels of proline only after prostate massage (P = 0,032) were detected. Higher levels of proline and homoserine and tyramine correlate with GS7 with respect to GS 6 and GS 5.
Fernandez-Peralbo et al. (2016) [69]	62 PrC patients 42 controls	Sample preparation: Thawing, centrifugation, pre-treatment with chemicals Analytical techniques: LC-QTOF	Derivatives of lysine, histidine, arginine, tyrosine, tryptophan, taurine, alanine, aspartate, glutamate, glutamine, purine, pyrimidine	Almost all metabolites were present at lower concentrations in PrC patients than in controls, Training: Specificity 92.9%; Sensibility 88.4% Validation: Specificity 78.6%; Sensibility 63.2%
Gkotsos et al. (2017) [60]	52 PrC patients 49 controls	Sample preparation: Thawing, centrifugation, pre-treatment with chemicals Analytical techniques: UPLC-MS/MS	Sarcosine; Uracil; Kynurenic acid	Decreased median sarcosine and kynurenic acid and increased uracil concentrations were observed for patients with prostate cancer compared to participants without malignancy.
Derezinski et al. (2017) [70]	49 PrC patients with different stages of disease 40 controls	Sample preparation: Thawing, centrifugation, pre-treatment with chemicals Analytical techniques: LC-ESI-MS/MS	1-methylhistidine 3-methylhistidine Alanine, Arginine, Argininosuccinic acid, Asparagine, Aspartic acid, Citrulline Carnosine	In PrC samples, taurine was present at significant higher level. The PLS-DA model built on selected metabolites achieved sensitivity and specificity of 89.47% and 73.33%, respectively, whereas the total group membership classification value was 82.35%.

**Table 4:** Literary studies investigating urinary colorectal cancer biomarkers.

Author (Year) [Ref]	Population	Experimental Method	Biomarkers	Results
Qiu et al. (2010) [73]	60 colorectal cancer (CrC) patients (42–76 years) at different stages 63 controls without any diseases or interferences	Sample preparation: Thawing, centrifugation and pre-treatment with chemicals Analytical techniques: GC-MS	Succinate; Isocitrate; Citrate; 5-hydroxytryptophan; 5-ydroxyindoleacetate; Tryptophan; Glutamate; 5-oxoproline; N-acetyl-aspartate; 3-methyl histidine; Histidine; p-cresol; 2-hydroxyhippurate; Phenylacetate; Phenylacetylglutamine; p-ydroxyphenylacetate	Considering preoperative CrC patients and healthy controls, levels of succinate, isocitrate, citrate, 3-methyl-histidine and histidine were lower in CrC patients than healthy patients. Levels of 5-hydroxytryptophane, 5-hydroxyindoleacetate, tryptophan, glutamate, 5-oxoproline, N-acetyl-aspartate, p-cresol, 2-hydroxyhippurate, phenylacetate, phenylacetylglutamine and p-hydroxyphenylacetate were higher in CrC patients than healthy ones. The experiments on rats indicate what are the biological mechanisms for the different metabolites’ beahaviour.
Chen et al. (2012) [76]	20 CrC patients (37–87 years) with tumor at different stages 14 controls without other diseases or interferences (50–86 years)	Sample preparation: Centrifugation and pre-treatments with chemicals Analytical techniques: CE-ESI-MS	Lactic acid; Arginine; Isoleucine; Leucine; Valine; Citric acid; Histidine; Methionine; Serine; Aspartic acid; Malic acid; Succinic acid	Levels of lactic acid, arginine, isoleucine, leucine and valine were higher in CrC patients. Levels of citric acid, histidine, methionine, serine, aspartate, malic acid and succinate were lower in CrC patients. The values of valine and isoleucine were lower in CrC patients at III–IV stages than those at I–II stages.
Cheng et al. (2012) [77]	101 CrC patients (24–83 years) at different stages 103 controls without other diseases or interferences (31–76 years)	Sample preparation: Centrifugation and pre-treatments with chemicals Analytical techniques: GC-TOFMS UPLC-QTOFMS	Citrate; Hippurate; p-cresol; 2-aminobutyrate; Myristate; Putrescine; Kynurenate	The levels of 2-aminobutyrate and putrescine are higher in CrC patients than healthy ones. The levels of citrate, hippurate, p-cresol, myristate and kynurenate are lower in CrC patients than healthy ones.

**Table 5:** Literary studies investigating urinary biomarkers for stomach cancer.

Authors (Year) [Ref]	Population	Experimental Method	Biomarkers	Results
Dong et al. (2009) [82]	144 gastric cancer (GC) patients (50–68 years) 144 controls (50–67 years)	Sample preparation: thawing of urine samples, addition with chemicals and extraction Analytical technique: LC-MS	Prostaglandin E2 metabolite (PGE-M)	The level of urinary PGE-M is higher in GC patients than controls.
Chen et al. (2014) [83]	26 GC patients at different stages 14 controls	Sample preparation: Pre-treatment with chemicals and centrifugation. Analytical technique: MRB-CE-MS	Arginine Leucine Isoleucine Valine Citric acid Succinate Histidine Methionine Serine Aspartate	Arginine. Leucine, Isoleucine and Valine were significantly higher in RCC patients with respect to controls, while citric acid, Histidine, Methionine, Serine, aspartate, malic acid and succinate were remarkably lower in RCC patients compared to controls. Moreover, Valine and Isoleucine levels differed in advanced stage RCC and early stage RCC (urine from early stage RCC patients were characterized by higher levels)
Jung et al. (2014) [79]	50 GC patients (38–81 years) at different stages 50 controls (38–78 years)	Sample preparation: thawing samples, centrifugation and addition with chemicals Analytical technique: H NMR	2-Oxobutyrate, 3-Aminoisobutyrate, 3-Indoxylsulfate, 4-Hydroxyphenylacetate, Acetate, Acetone, Alanine, Arginine, Betaine, Formate, Glycine, Glycolate, Histidine, Lactate, Leucine, Mannitol, Methionine, N-Methylhydantoin, O-Acetylcarnitine, Phenylacetylglycine, Phenylalanine, Putrescine, Succinate, Taurine, Tyrosine and Valine,1-Methylnicotinamide, Hypoxanthine	2-Oxobutyrate, 3-Aminoisobutyrate, 3-Indoxylsulfate, 4-Hydroxyphenylacetate, Acetate, Acetone, Alanine, Arginine, Betaine, Formate, Glycine, Glycolate, Histidine, Lactate, Leucine, Mannitol, Methionine, N-Methylhydantoin, O-Acetylcarnitine, Phenylacetylglycine, Phenylalanine, Putrescine, Succinate, Taurine, Tyrosine and Valine levels are higher in GC patients than controls. 1-Methylnicotinamide and Hypoxanthine levels are lower in GC patients than controls.
Chan et al. (2016) [78]	43 GC patients (53–77 years) at different stages 40 controls (54–72 years) 40 resemble benign (BN) (54–72 years)	Sample preparation: thawing, addition with chemicals and centrifugation Analythical technique: H NMR	2-hydroxyisobutyrate 3-indoxylsulfate Alanine	Among the 25 metabolites investigated, only 2-hydroxyisobutyrate, 3-indoxylsulfate and alanine proved to provide useful information for diagnostic purposed and were considered to build the discrimination model, achieving a diagnostic accuracy of 95%.

**Table 6:** Literary studies investigating urinary liver cancer biomarkers.

Authors (Year) [Ref]	Population	Experimental Method	Biomarkers Proposed	Results
Wu et al. (2009) [89]	20 hepatic cancer (HC) patients (30–53 years) 20 controls (35–58 years) All studied groups were males.	Sample preparation: centrifugation, addition of chemicals and evaporation. Analytical technique: GC-MS	glycine; octanedioic acid; tyrosine; threonine and butanedioic acid heptanedioic acid; ethanedioic acid; xylitol; urea; phosphate; propanoic acid; primidine; butanoic acid; trihydroxypentanoic acid; hypoxanthine; arabinofuranose; hydroxy proline dipeptid; xylonic acid	The levels of glycine, octanedioic acid, tyrosine, threonine and butanedioic acid are higher in HC patients than healthy ones. The levels of heptanedioic acid, ethanedioic acid, xylitol, urea, phosphate, propanoic acid, primidine, butanoic acid, trihydroxypentanoic acid, hypoxanthine, arabinofuranose, hydroxy proline dipeptid and xylonic acid are lower in HC patients than in control ones.
Chen et al. (2011) [84]	82 HC patients (29–76 years) at different stages 71 controls (42–65 years) 24 benign liver tumor patients (18–65 years) as hemangioma, focal nodular hyperplasia of liver, liver cirrhosis, liver cyst, intrahepatic bile duct stone and recurrent hemangioma after surgery	Sample preparation: centrifugation, pre-treatments with chemicals and drying Analytical technique: GC-TOFMS UPLC-QTOFMS	Glycocholic acid; cysteine; tyrosine; phenylalanine; dopamine; adenosine; uric acid; xanthine; hypoxanthine; hypotaurine; taurine; 5-Hydroxy-tryptophan; N-Acetyl-L-aspartic acid; pyridoxal; threonine; dihydrouracil; agmatine; O-Phospho-L-serine; N-Acetyl-neuraminic acid4-Hydroxyphenylacetate; trimethylamine N-oxide; cysteine; alanine; homovanillate; normetanephrine; adenine; cysteic acid; nicotinic acid; succinic acid; carnosine; 2-pyrrolidone-5-carboxylic acid; 6-aminocaproic acid; creatine	Glycocholic acid, cystine, tyrosine, phenylalanine, dopamine, adenosine, uric acid, xanthine, hypoxanthine, hypotaurine, taurine, 5-Hydroxy-tryptophan, N-Acetyl-L-aspartic acid, pyridoxal, threonine, dihydrouracil, agmatine, O-Phospho-L-serine and N-Acetyl-neuraminic acid levels in HC patients are higher than healthy ones. 4-Hydroxyphenylacetate, trimethylamine N-oxide, cysteine, alanine, homovanillate, normetanephrine, adenine, cysteic acid, nicotinic acid, succinic acid, carnosine, 2-pyrrolidone-5-carboxylic acid, 6-aminocaproic acid, creatine levels in HC patients are lower than in healthy ones. Trimethylamine N-oxide, alanine, homovanillate, normetanephrine, cysteic acid, 6-Aminocaproic acid, creatine and 5-hydroxylysine levels in HC patients at I-II stages are lower than healthy ones. Glycocholic acid, cystine, homoserine, tyramine, tyrosine, dopamine, adenosine, xanthine, hypoxanthine, hypotaurine, 5-Hydroxy-tryptophan, N-Acetyl-L-Aspartic acid, threonine, dihydrouracil, ethymalonic acid, agmatine and N-Acetyl-neuraminic acid levels in HC patients at III-IV stages are higher than healthy ones. Trimethylamine N-oxide, cysteine, adenine, cysteic acid, citrulline, 6-Aminocaproic acid, creatine levels in HC patients at III-IV stages are lower than healthy ones.
Osman et al. (2016) [90]	55 HC patients (41–58 years) 40 LC patients (40–59 years) with HCV infection 45 controls (39–57 years) All the studied groups were males.	Sample preparation: centrifugation and pre-treatments with chemicals. Analytical technique: GC-MS.	Glycine Serine Threonine Proline Citric acid Phosphate Pyrimidine Arabinose Xylitol Hippuric acid Xylonic acid Glycerol	Glycine, serine, threonine, proline, and citric acid levels in HC patients are higher than healthy ones. Urea, phosphate, pyrimidine, arabinose, xylitol, hippuric acid, xylonic acid and glycerol levels in HC patients are lower than control ones.
Shariff et al. (2016) [91]	13 HC patients (29–82 years) at different stages 25 LC patients (28–79 years) at different stages No controls involved	Sample preparation: Pre-treatments with chemicals and centrifugation. Analytical technique: HNMR spectroscopy	Carnitine Formate Ciitrate doublet Hippurate p-cresol sulfate Creatinine methyl Creatinine methylene	Carnitine and formate levels in HC patients are higher than liver cirrhosis patients. Citrate doublet, hippurate, p-cresol sulfate, creatinine methyl and creatinine methylene levels in HC patients are lower than liver cirrhosis patients.

**Table 7:** Literary studies investigating urinary biomarkers for bladder cancer.

Authors(Year) [Ref]	Population	Experimental Method	Biomarkers	Results
Jin et al. (2014) [92]	138 Bladder cancer (BlC) patients at different tumor stages (53–78 years) 121 controls (55–73 years): 69 healthy people 52 patients with hematuria due to non-malignant disease	Sample preparation: thawing of urine sample, centrifugation and treatments with chemicals Analytical technique: GC-MS	Succinate Pyruvate Oxoglutarate Carnitine Phosphoenolpyruvate Trimethyllysine Melatonin Isovalerylcarnitine Glutarylcarnitine Octenoylcarnitine Decanoylcarnitine Acetyl-CoA Carnitine palmitoyltransferase Carnitine acylcarnitine translocaselike protein Dihydrolipoyl dehydrogenase	The levels of succinate, pyruvate, oxoglutarate, carnitine, phosphoenolpyruvate, trimethyllysine, isovalerylcarnitine, octenoylcarnitine, acetyl-CoA, carnitine palmitoyltransferase and carnitine acylcarnitine translocaselike protein were found to be higher in BlC patients than controls The levels of melatonin, glutarylcarnitine, decanoylcarnitine and dihydrolipoyl dehydrogenase were found to be lower in BlC patients than controls
Nakai et al. (2015) [95]	61 BlC patients with different stage of tumor (34–91 years) 50 controls (25–92 years) with no cancer-related findings	Sample preparation: thawing, centrifugation and treatments with chemicals Analitycal technique: spectrophotometer	Protoporphyrin IX	There are a lot of differences in protoporphyrin IX between BlC patients and controls. These differences are present in BlC patients with different tumor stages and between MIBC patients and NMIBC patients too.
Alberice et al. (2013) [93]	48 BlC patients at different stages	Sample preparation: centrifugation and treatments with chemicals Analytical technique: CE-TOF-MS LC-QTOF-MS	Betaine; Leucine; Hypoxanthine; Hystidine; Phenylalanine; Uric acid; 1-Methylhistidine Nε,Nε,Nε-trimethyllysiine; Nε,Nε-dimethyllysine; Tyrosine; Galacticol7sorbitol/mannitol; 3-Amino-2-naphthoic acid; Dopaquinone; Acetylcarnitine; Tryptophan; Carnosine; 2,6,10-Trimethyl undecanoic acid; Cystine N-acetyltryptophan; Palmitic amide Heptanoylcarnitine; 12S-hydroxyoctadienoic acid, Decanoylcarnitine; 6-Keto-decanoyl carnitine	Hystidine, phenylalanine, tyrosine and tryptophan levels are higher in BlC patients than controls. Tryptophan is significant in low risk patients, so important for detection at early stage. Hystidine and tyrosiine are higher in high-risk patients with respect to low-risk patients, while N-acetyltryptophan, leucine, hypoxanthine and uric acid levels are higher in low risk patients. Dopaquinone, Nε,Nε,Nε-trimethyllysiine, Nε,Nε-dimethyllysine and carnine derivatives concentrations are higher in patients with recurrence of the disease.
Huang et al. (2011) [94]	27 BlC patients: (42–71 years) at different stages 32 controls (46–67 years)	Sample preparation: thawing, centrifugation, treatment with chemicals and filtration through cellulose filters Analytical technique: HPLC-MS with two different columns	Octenoylcarnitine (carnitine C8:1), Carnitine C9:1, 9-Decenoylcarnitine (carnitine C10:1), Acetyl-carnitine, 2,6-dimethylheptanoyl carnitine, Hippuric acid	The level of carnitine C8:1, carnitine C9:1, carnitine C10:1,2,6-dimethylheptanoyl carnitine and hippuric acid is lower in BlC patients than controls, while the level of acetyl-carnitine is higher in BlC patients than controls.
Cauchi et al. (2016) [96]	72 BlC patients (56–88 years) at different stages 205 controls: (18- 89 years)	Sample preparation: thawing, treatments with chemicals and extraction on a carbon/PDMS fiber Analitycal technique: GC-TOF-MS	2-pentanone; 2;3-butanedione; 4-heptanone; Dimethyl disulphide; Hexanal; Benzaldehyde; Butyrophenone; 3-hydroxyanthranilic acid; Benzoic acid; trans-3-hexanoic acid; cis-3-hexanoic acid; 2-Butanone; 2-propanol Acetic acid; Piperitone; Thujone	2-pentanone, 2,3-butanedione, 4-heptanone, dimethyl disulphide, 2-Butanone, 2-propanol, acetic acid, piperitone and thujone levels are lower in BlC patients than controls. Hexanal, benzaldehyde, butyrophenone,3-hydroxyanthranilic acid, benzoic acid, trans-3-hexanoic acid and cis-3-hexanoic acid levels are higher in BlC patients than controls.

**Table 8:** Literary studies investigating urinary pancreatic cancer biomarkers.

Authors (Year) [Ref]	Population	Experimental Method	Biomarkers	Results
Napoli et al. (2012) [116]	33 pancreatic cancer (PaC) patients (56–68 years) 54 controls (55–67 years)	Sample preparation: thawing samples, addition of chemicals and centrifugation Analytical technique: H-NMR	Acetoacetate; Acetylated compounds; Adenine; Alanine; Bile salts; Citrate; Creatinine; Formate; Glucose; Glycine; Hippurate; 2-hydroxyisobutyrate; 3-hydroxyisovalerate; 4-hydroxyphenylacetate; Isobutyrate; Lactate; Leucine; Dimethylamine; Trimethylamine-N-oxide; 3-methylhistidine; 1-methylnicotinamide; 2-phenylacetamide; Trigonelline; Valine	The level of acetoacetate, acetylated compounds, glucose, leucine and 2-phenylacetamide is higher in PC patients than in controls. The level of citrate, creatinine, glycine, hippurate, 3-hydroxyisovalerate and trigonelline is lower in PaC patients than in controls.
Davis et al. (2013) [117]	32 PaC patients (48–83 years) at different stages 25 benign pancreatitis patients (42–77 years) 32 controls (47–84 years)	Sample preparation: thawing samples and addition with chemicals Analytical technique: H-NMR	Acetone; Hypoxanthine; O-Acetylcarnitine; Dimethylamine; Choline; 1-Methylnicotinamide; Threonine; Fucose; Cis-Aconitate; 4-Pyridoxate; Glucose; Trimethylamine-N-oxide; Aminobutyrate; Tryptophan; Trigonelline; Xylose; Trans-Aconitate; Methanol; 4-Hydroxyphenylacetate; 2-Hydroxyisobutyrate; Taurine	The level of acetone, hypoxanthine, O-Acetylcarnitine, dimethylamine, choline, 1 Methylnicotinamide, threonine, fucose, cis-Aconitate, 4-Pyridoxate, glucose, trimethylamine-N-oxide, aminobutyrate, tryptophan, xylose, trans-Aconitate, 4-Hydroxyphenylacetate, 2 Hydroxyisobutyrate and taurine is higher in PaC patients than in controls. The level of trigonelline and methanol is lower in PaC patients than in controls.
Lusczek et al. (2015) [118]	5 PaC patients (42–63 years) at different stages 92 chronic pancreatitis patients (42–77 years) 87 controls (24–62 years)	Sample preparation: thawing samples, addition with chemicals and centrifugation Analytical technique: NMR	Adenosine; citrate	The level of citrate is lower in PaC patients than in controls.
Radon et al. (2015) [119]	192 PaC patients at different stages 92 chronic pancreatitis patients 87 controls	Sample preparation: pre-treatments with chemical and extraction Analytical technique: GeLC/MS/MS ELISA	LYVE1; REG1A; TFF1	LYVE1, REG1A and TFF1 levels were significantly higher in PaC patients with respect to controls. The LYVE1, REG1A and TFF1 levels increase with tumor stage, allowing the discrimination between early and late PaC.
Mayerle et al. (2017) [120]	271 PaC patients at different stages 282 chronic pancreatitis patients 100 liver cirrhosis 261 controls	Sample preparation: pre-treatments with chemical and extraction Analytical technique: GeLC/MS/MS ELISA	histidine, proline, sphingomyelin d18:2, sphingomyelin d17:1, phosphatidylcholine, isocitrate, sphingagine-1-phosphate, pyruvate, and ceramide	The model based on those 9 metabolites and CA19–9 achieved a diagnostic accuracy of 96%.

**Table 9:** Literary studies investigating urinary renal cancer biomarkers.

Authors (Year) [Ref]	Population	Experimental Method	Biomarkers Proposed	Results
Han et al. (2005) [126]	42 renal or kidney cancer (RC) patients 30 controls 10 PCa patients	Sample preparation: Centrifugation and pre-treatment with chemicals Analytical technique: ELISA	human kidney injury molecule-1 (hKIM-1)	hKIM-1 levels in urine were significantly higher in patients with RC (0.39 ± 0.06 ng/mgUcr) compared with levels in urine from PCa patients (0.12 ± 0.03 ng/mgUcr) or normal control subjects (0.05 ± 0.01 ng/mgUcr).
Bosso et al. (2008) [127]	39 RC patients (52–88 years) at different stages 29 controls (44–86 years)	Sample preparation: Centrifugation and pre-treatment with chemicals Analytical technique: MALDI-TOF	Three different fragments of uromodulin (A, B and C)	Diagnostic accuracy of biomarkers A and B was above 90%, while the diagnostic accuracy of biomarker C was of 84%. The model built considering all fragments achieved showed better performance in classifying RC patients and controls (training: specificity 100% and sensitivity 95%; test: specificity 100% and sensitivity 85%).
Ganti et al. (2011) [128]	29 RC patients at different stages 33 controls	Sample preparation: Addition of chemicals and centrifugation. Analytical technique: Untargeted metabolic analysis GC-MS	Isobutyrylcarnitine Suberoylcarnitine Acetylcarnitine	Isobutyrylcarnitine, Suberoylcarnitine and Acetylcarnitine levels were higher in RC patients than in controls. Acylcarnitines levels in urine increased as function of tumor grade.

**Table 10:** Literary studies investigating urinary testicular cancer biomarkers.

Authors (Year) [Ref]	Population	Experimental Method	Biomarkers	Results
Lipsett et al. (1966) [133]	1 testicular cancer (TC) patient	Sample preparation: Collection of 24 h urine and pre-treatment with chemicals Analytical techniques: GC-MS	17-ketosteroid 17-hydroxycorticoids	Monitoring of response to cancer treatments
Eyben (1978) [134]	27 TC patients with different stages of disease 18 controls (15–50 years)	Sample preparation: Collection of 24 h urine Analytical techniques: Extractor, radioimmunoassay, fluorimetric method	Human chorionic gonadotropin HCG	Higher HCG levels in sick patients (above 300 IC/24 h) Correlation between post-operative HCG and prognosis

**Table 11:** Concentration levels of recurrent cancer biomarkers in urine samples from cancer patients with respect to controls (> higher concentrations; < lower concentrations; nd: no information about concentrations; - no correlation).

Recurrent Cancer Biomarkers in Urine	Concentration Levels in Urine from Cancer Patients with Respect to Controls									
	LC	BC	PrC	CrC	GC	HC	BlC	PaC	RC	TC
Glycine	-	-	-	-	>	>	-	<	-	-
Serine	-	-	-	<	<	>	-	-	-	-
Threonine	-	-	>	>	-	>	-	>	-	-
Alanine	-	-	nd	-	>	<	-	nd	-	-
Phenylalanine	>	-	<	-	>	>	>	-	-	-
Tyrosine	-	-	>	-	>	>	>	-	-	-
Hippurate	-	-	-	<	-	<	-	<	-	-
Hydroxyhippurate	-	-	-	>	-	-	-	-	-	-
Tryptophan	-	-	-	>	-	-	>	>	-	-
Kynurenate	-	-	-	<	-	-	-	-	-	-
Lactate	-	-	-	-	>	-	-	nd	-	-
Lactic acid	-	-	-	-	>	-	-	-	-	-
Indoleacetate	-	nd	-	>	-	-	-	-	-	-
Taurine	>	-	>	-	>	>	-	>	-	-
Hypotaurine	-	-	-	-	-	>	-	-	-	-
Citrate	-	-	-	<	-	<	-	<	-	-
Isocitrate	-	-	-	<	-	-	-	-	-	-
Putrescine	-	-	nd	>	>	-	-	-	-	-
Succinate	-	<	-	<	>/<	-	>	-	-	-
Aconitate	-	-	nd	-	-	<	-	>	-	-
Citrulline	-	-	nd	-	-	<	-	-	-	-
Valine	>	<	-	>	>	-	-	nd	-	-
Leucine	-	<	>/<	>	>	-	>	>	-	-
Isoleucine	-	>	>	>	>	-	-	-	-	-
Arginine	-	-	>	>	>	-	-	-	-	-
Creatinine	-	<	>	-	-	<	-	<	-	-
Adenosine	-	-	-	-	-	<	-	<	-	-
Uridine	-	>	-	-	-	-	-	-	-	-
Carnitine	>	-	-	-	-	>	>/<	-	>	-
Purine	-	-	nd	-	-		-	-	-	-
Xanthine	-	-	-	-	-	<	-	-	-	-
Adenine	-	-	-	-	-	<	-	nd	-	-
Guanosine	-	>	-	-	-		-	-	-	-
Xanthine	-	-	-	-	-	<	-	-	-	-
Aspartic acid	-	-	>	nd	-		-	-	-	-
Malic acid	-	-	-	<	-	<	-	-	-	-
Succinic acid	-	-	nd	nd	-	<	-	-	-	-
Xylonic acid	-	-	>	-	-	<	-	-	-	-
Kynureic acid	-	-	<	-	-	-	-	-	-	-
Octanedionic acid	-	-	-	-	-	>	-	-	-	-
Butanedionic acid	-	-	-	-	-	>	-	-	-	-
Heptanedionic acid	-	-	-	-	-	<	-	-	-	-
Ethanedioic acid	-	-	-	-	-	<	-	-	-	-
Propanoic acid	-	-	-	-	-	<	-	-	-	-
Butanoic acid	-	-	>	-	-	<	-	-	-	-
Trihydroxypentanoic acid	-	-	<	-	-	-	-	-	-	-
Glicholic acid	-	-	-	-	-	>	-	-	-	-
Uric acid	-	-	>	-	-	<	>	-	-	-
Citric acid	-	-	-	<	<	>	-	-	-	-
Nicotinic acid	-	-	-	-	-	<	-	-	-	-
Hippuric acid	>	-	-	-	-	<	<	-	-	-
Acetic acid	-	-	-	-	-	-	<	-	-	-

**Table 12:** Odor properties of recurrent biomarkers for different cancer forms.

Recurrent Urinary Cancer Biomarker	OT (ppmouE/m3) [151,152]	Odor Description [152]	Cancer Type
Acetic acid	0.004–204	Sour, pungent, vinegar	BlC
Succinic acid	-	Pungent	CrC, PrC, HC
Diethylamine and derivatives	0.0033–14.3	Musty, fishy, amine	CrC, PrC, TC, HC, BC, HC
Trimethylamine and derivatives	0.00002–1.82	Fishy, pungent	HC
Pyridine	0.01–12	Burnt, pungent, nauseating	PrC
Cresol (all isomers)	0.00005–0.009	Phenol, irritating, smoky, empyreumatic, burnt plastic	CrC, HC
Phenol	0.0045–1.95	Acid	BC
Cyclohexanone	0.052–219	Sweet, sharp	LC
L-cysteine	24.2	Sulphur, rotten eggs	PrC, HC
D-cysteine	26.7	Sulphur, rotten eggs	PrC, HC
L-methionine	11.9	Moldy, rotten dairy products	CrC, PrC, GC
D-methionine	1.5	Moldy, rotten dairy products	CrC, PrC, GC
L-proline	11,513	Chlorine, semen, sperm	CrC, PrC, HC, LC
D-proline	8635	Chlorine, semen, sperm	CrC, PrC, HC, LC
Histidine	-	Slightly bitter acid	CrC, PrC, BlC, GC
Arginine	-	Bitter	CrC, PrC, GC
Glycine	-	Sweet, refreshing	PrC, HC, GC
Tyrosine	-	Soft, flat, stale	PrC, HC, BlC, GC
Indole	21–140	Fecal	CrC, PrC, BC

## References

[1] Bray F., Ferlay J., Soerjomataram I., Siegel R.L., Torre L.A., Jemal A. (2018). Global cancer statistics 2018: Globocan estimates of incidence and mortality worldwide for 36 cancers in 185 countries. CA Cancer J. Clin..

[2] Fidler M.M., Bray F., Soerjomataram I. (2018). The global cancer burden and human development: A review. Scand. J. Public Health.

[3] Richards M.A. (2009). The size of the prize for earlier diagnosis of cancer in england. Br. J. Cancer.

[4] Bax C., Taverna G., Eusebio L., Sironi S., Grizzi F., Guazzoni G., Capelli L. (2018). Innovative diagnostic methods for early prostate cancer detection through urine analysis: A review. Cancers.

[5] Di Lena M., Porcelli F., Altomare D.F. (2016). Volatile organic compounds as new biomarkers for colorectal cancer: A review. Colorectal Dis..

[6] Das V., Kalita J., Pal M. (2017). Predictive and prognostic biomarkers in colorectal cancer: A systematic review of recent advances and challenges. Biomed. Pharmacother..

[7] Capelli L., Taverna G., Bellini A., Eusebio L., Buffi N., Lazzeri M., Guazzoni G., Bozzini G., Seveso M., Mandressi A. (2016). Application and uses of electronic noses for clinical diagnosis on urine samples: A review. Sensors.

[8] Asimakopoulos A.D., Del Fabbro D., Miano R., Santonico M., Capuano R., Pennazza G., D’Amico A., Finazzi-Agro E. (2014). Prostate cancer diagnosis through electronic nose in the urine headspace setting: A pilot study. Prostate Cancer Prostatic Dis..

[9] de Meij T.G., Larbi I.B., van der Schee M.P., Lentferink Y.E., Paff T., Terhaar Sive Droste J.S., Mulder C.J., van Bodegraven A.A., de Boer N.K. (2014). Electronic nose can discriminate colorectal carcinoma and advanced adenomas by fecal volatile biomarker analysis: Proof of principle study. Int. J. Cancer.

[10] Kort S., Brusse-Keizer M., Schouwink H., De Jongh F., Citgez E., Gerritsen J.W., Van Der Palen J. (2018). Detection of small cell lung cancer by electronic nose. Eur. Respir. J..

[11] Peng G., Hakim M., Broza Y.Y., Billan S., Abdah-Bortnyak R., Kuten A., Tisch U., Haick H. (2010). Detection of lung, breast, colorectal, and prostate cancers from exhaled breath using a single array of nanosensors. Br. J. Cancer.

[12] Lippi G., Plebani M. (2019). Diabetes alert dogs: A narrative critical overview. Clin. Chem. Lab. Med..

[13] Jadoon S., Karim S., Akram M.R., Kalsoom Khan A., Zia M.A., Siddiqi A.R., Murtaza G. (2015). Recent developments in sweat analysis and its applications. Int. J. Anal. Chem..

[14] Bosch S., Berkhout D.J., Ben Larbi I., de Meij T.G., de Boer N.K. (2019). Fecal volatile organic compounds for early detection of colorectal cancer: Where are we now?. J. Cancer Res. Clin. Oncol..

[15] Rooney N.J., Guest C.M., Swanson L.C.M., Morant S.V. (2019). How effective are trained dogs at alerting their owners to changes in blood glycaemic levels? Variations in performance of glycaemia alert dogs. PLoS ONE.

[16] Elliker K.R., Sommerville B.A., Broom D.M., Neal D.E., Armstrong S., Williams H.C. (2014). Key considerations for the experimental training and evaluation of cancer odour detection dogs: Lessons learnt from a double-blind, controlled trial of prostate cancer detection. BMC Urol..

[17] Gordon R.T., Schatz C.B., Myers L.J., Kosty M., Gonczy C., Kroener J., Tran M., Kurtzhals P., Heath S., Koziol J.A. (2008). The use of canines in the detection of human cancers. J. Altern. Complement. Med. (New York, NY).

[18] Bernabei M., Pennazza G., Santonico M., Corsi C., Roscioni C., Paolesse R., Di Natale C., D’Amico A. (2008). A preliminary study on the possibility to diagnose urinary tract cancers by an electronic nose. Sens. Actuators B Chem..

[19] Cornu J.N., Cancel-Tassin G., Ondet V., Girardet C., Cussenot O. (2011). Olfactory detection of prostate cancer by dogs sniffing urine: A step forward in early diagnosis. Eur. Urol..

[20] Fischer-Tenhagen C., Johnen D., Nehls I., Becker R. (2018). A proof of concept: Are detection dogs a useful tool to verify potential biomarkers for lung cancer?. Front. Vet. Sci..

[21] Shirasu M., Touhara K. (2011). The scent of disease: Volatile organic compounds of the human body related to disease and disorder. J. Biochem..

[22] Marimuthu A., O’Meally R.N., Chaerkady R., Subbannayya Y., Nanjappa V., Kumar P., Kelkar D.S., Pinto S.M., Sharma R., Renuse S. (2011). A comprehensive map of the human urinary proteome. J. Proteome Res..

[23] Hammoudi N., Ahmed K.B.R., Garcia-Prieto C., Huang P. (2011). Metabolic alterations in cancer cells and therapeutic implications. Chin. J. Cancer.

[24] Sciacovelli M., Gaude E., Hilvo M., Frezza C. (2014). The metabolic alterations of cancer cells. Methods Enzymol..

[25] Jang M., Kim S.S., Lee J. (2013). Cancer cell metabolism: Implications for therapeutic targets. Exp. Amp Mol. Med..

[26] Taverna G., Tidu L., Grizzi F. (2015). Sniffing out prostate cancer: A new clinical opportunity. Cent. Eur. J. Urol..

[27] Cameron S.J., Lewis K.E., Beckmann M., Allison G.G., Ghosal R., Lewis P.D., Mur L.A. (2016). The metabolomic detection of lung cancer biomarkers in sputum. Lung Cancer (Amsterdam, Netherlands).

[28] Cancer Research UK Lung Cancer: Stages, Types and Grades. https://www.cancerresearchuk.org/about-cancer/lung-cancer/stages-types-grades/types.

[29] Wehinger A., Schmid A., Mechtcheriakov S., Ledochowski M., Grabmer C., Gastl G.A., Amann A. (2007). Lung cancer detection by proton transfer reaction mass-spectrometric analysis of human breath gas. Int. J. Mass Spectrom..

[30] Gessner C., Rechner B., Hammerschmidt S., Kuhn H., Hoheisel G., Sack U., Ruschpler P., Wirtz H. (2010). Angiogenic markers in breath condensate identify non-small cell lung cancer. Lung Cancer (Amsterdam, Netherlands).

[31] Zou Y., Wang L., Zhao C., Hu Y., Xu S., Ying K., Wang P., Chen X. (2013). Cea, scc and nse levels in exhaled breath condensate--possible markers for early detection of lung cancer. J. Breath Res..

[32] Brussino L., Culla B., Bucca C., Giobbe R., Boita M., Isaia G., Heffler E., Oliaro A., Filosso P., Rolla G. (2014). Inflammatory cytokines and vegf measured in exhaled breath condensate are correlated with tumor mass in non-small cell lung cancer. J. Breath Res..

[33] Yang Q., Shi X., Wang Y., Wang W., He H., Lu X., Xu G. (2010). Urinary metabonomic study of lung cancer by a fully automatic hyphenated hydrophilic interaction/rplc-ms system. J. Sep. Sci..

[34] Guadagni R., Miraglia N., Simonelli A., Silvestre A., Lamberti M., Feola D., Acampora A., Sannolo N. (2011). Solid-phase microextraction-gas chromatography-mass spectrometry method validation for the determination of endogenous substances: Urinary hexanal and heptanal as lung tumor biomarkers. Anal. Chim. Acta.

[35] Hanai Y., Shimono K., Matsumura K., Vachani A., Albelda S., Yamazaki K., Beauchamp G.K., Oka H. (2012). Urinary volatile compounds as biomarkers for lung cancer. Biosci. Biotechnol. Biochem..

[36] American Camcer Society How Common Is Breast Cancer?. https://www.cancer.org/cancer/breast-cancer/about/how-common-is-breast-cancer.html.

[37] Sree S.V., Ng E.Y.K., Acharya R.U., Faust O. (2011). Breast imaging: A survey. World J. Clin. Oncol..

[38] McCready T., Littlewood D., Jenkinson J. (2005). Breast self-examination and breast awareness: A literature review. J. Clin. Nurs..

[39] Kalager M., Zelen M., Langmark F., Adami H.O. (2010). Effect of screening mammography on breast-cancer mortality in norway. N. Engl. J. Med..

[40] Berry D.A., Cronin K.A., Plevritis S.K., Fryback D.G., Clarke L., Zelen M., Mandelblatt J.S., Yakovlev A.Y., Habbema J.D.F., Feuer E.J. (2005). Effect of screening and adjuvant therapy on mortality from breast cancer. N. Engl. J. Med..

[41] Bleyer A., Welch H.G. (2012). Effect of three decades of screening mammography on breast-cancer incidence. N. Engl. J. Med..

[42] Jackman R.J., Marzoni F.A., Rosenberg J. (2009). False-negative diagnoses at stereotactic vacuum-assisted needle breast biopsy: Long-term follow-up of 1,280 lesions and review of the literature. Am. J. Roentgenol..

[43] Imagins Benefits and Risks of Breast Biopsy. http://www.imaginis.com/biopsy/benefits-and-risks-of-breast-biopsy-4.

[44] Fernández C.A., Yan L., Louis G., Yang J., Kutok J.L., Moses M.A. (2005). The matrix metalloproteinase-9/neutrophil gelatinase-associated lipocalin complex plays a role in breast tumos growth and is present in the urine of breast cancer patients. Hum. Cancer Biol..

[45] Pories S.E., Zurakowski D., Roy R., Lamb C.C., Raza S., Exarhopoulos A., Scheib R.G., Schumer S., Lenahan C., Borges V. (2008). Urinary metalloproteinases: Noninvasive biomarkers for breast cancer risk assessment. Cancer Epidemiol. Biomark. Prev..

[46] Chung B.C., Lee D., Nam H., Lee K., Kim Y. (2009). Combining tissue transcriptomics and urine metabolomics for breast cancer biomarker identification. Bioinformatics.

[47] Woo H.M., Kim K.M., Choi M.H., Jung B.H., Lee J., Kong G., Nam S.J., Kim S., Bai S.W., Chung B.C. (2009). Mass spectrometry based metabolomic approaches in urinary biomarker study of women’s cancers. Clin. Chim. Acta Int. J. Clin. Chem..

[48] Slupsky C.M., Steed H., Wells T.H., Dabbs K., Schepansky A., Capstick V., Faught W., Sawyer M.B. (2010). Urine metabolite analysis offers potential early diagnosis of ovarian and breast cancers. Clin. Cancer Res..

[49] Silva C.L., Passos M., Camara J.S. (2012). Solid phase microextraction, mass spectrometry and metabolomic approaches for detection of potential urinary cancer biomarkers—A powerful strategy for breast cancer diagnosis. Talanta.

[50] Siegel R.L., Miller K.D., Jemal A. (2018). Cancer statistics, 2018. CA Cancer J. Clin..

[51] Lee D.J., Mallin K., Graves A.J., Chang S.S., Penson D.F., Resnick M.J., Barocas D.A. (2017). Recent changes in prostate cancer screening practices and epidemiology. J. Urol..

[52] Harvey P., Basuita A., Endersby D., Curtis B., Iacovidou A., Walker M. (2009). A systematic review of the diagnostic accuracy of prostate specific antigen. BMC Urol..

[53] Kryvenko O.N., Epstein J.I. (2016). Prostate cancer grading: A decade after the 2005 modified gleason grading system. Arch. Pathol. Lab. Med..

[54] Loeb S., Vellekoop A., Ahmed H.U., Catto J., Emberton M., Nam R., Rosario D.J., Scattoni V., Lotan Y. (2013). Systematic review of complications of prostate biopsy. Eur. Urol..

[55] Rigau M., Olivan M., Garcia M., Sequeiros T., Montes M., Colás E., Llauradó M., Planas J., De Torres I., Morote J. (2013). The present and future of prostate cancer urine biomarkers. Int. J. Mol. Sci..

[56] Nam R.K., Saskin R., Lee Y., Liu Y., Law C., Klotz L.H., Loblaw D.A., Trachtenberg J., Stanimirovic A., Simor A.E. (2010). Increasing hospital admission rates for urological complications after transrectal ultrasound guided prostate biopsy. J. Urol..

[57] Sreekumar A., Poisson L.M., Rajendiran T.M., Khan A.P., Cao Q., Yu J., Laxman B., Mehra R., Lonigro R.J., Li Y. (2009). Metabolomic profiles delineate potential role for sarcosine in prostate cancer progression. Nature.

[58] Jiang Y., Cheng X., Wang C., Ma Y. (2010). Quantitative determination of sarcosine and related compounds in urinary samples by liquid chromatography with tandem mass spectrometry. Anal. Chem..

[59] Jentzmik F., Stephan C., Miller K., Schrader M., Erbersdobler A., Kristiansen G., Lein M., Jung K. (2010). Sarcosine in urine after digital rectal examination fails as a marker in prostate cancer detection and identification of aggressive tumours. Eur. Urol..

[60] Gkotsos G., Virgiliou C., Lagoudaki I., Sardeli C., Raikos N., Theodoridis G., Dimitriadis G. (2017). The role of sarcosine, uracil, and kynurenic acid metabolism in urine for diagnosis and progression monitoring of prostate cancer. Metabolites.

[61] Wu H., Liu T., Ma C., Xue R., Deng C., Zeng H., Shen X. (2011). Gc/ms-based metabolomic approach to validate the role of urinary sarcosine and target biomarkers for human prostate cancer by microwave-assisted derivatization. Anal. Bioanal. Chem..

[62] Stabler S., Koyama T., Zhao Z., Martinez-Ferrer M., Allen R.H., Luka Z., Loukachevitch L.V., Clark P.E., Wagner C., Bhowmick N.A. (2011). Serum methionine metabolites are risk factors for metastatic prostate cancer progression. PLoS ONE.

[63] Bianchi F., Dugheri S., Musci M., Bonacchi A., Salvadori E., Arcangeli G., Cupelli V., Lanciotti M., Masieri L., Serni S. (2011). Fully automated solid-phase microextraction-fast gas chromatography-mass spectrometry method using a new ionic liquid column for high-throughput analysis of sarcosine and n-ethylglycine in human urine and urinary sediments. Anal. Chim. Acta.

[64] Shamsipur M., Naseri M.T., Babri M. (2013). Quantification of candidate prostate cancer metabolite biomarkers in urine using dispersive derivatization liquid-liquid microextraction followed by gas and liquid chromatography-mass spectrometry. J. Pharm. Biomed. Anal..

[65] Heger Z., Cernei N., Gumulec J., Masarik M., Eckschlager T., Hrabec R., Zitka O., Adam V., Kizek R. (2014). Determination of common urine substances as an assay for improving prostate carcinoma diagnostics. Oncol. Rep..

[66] Khalid T., Aggio R., White P., De Lacy Costello B., Persad R., Al-Kateb H., Jones P., Probert C.S., Ratcliffe N. (2015). Urinary volatile organic compounds for the detection of prostate cancer. PLoS ONE.

[67] Tsoi T.H., Chan C.F., Chan W.L., Chiu K.F., Wong W.T., Ng C.F., Wong K.L. (2016). Urinary polyamines: A pilot study on their roles as prostate cancer detection biomarkers. PLoS ONE.

[68] Sroka W.D., Boughton B.A., Reddy P., Roessner U., Slupski P., Jarzemski P., Dabrowska A., Markuszewski M.J., Marszall M.P. (2017). Determination of amino acids in urine of patients with prostate cancer and benign prostate growth. Eur. J. Cancer Prev..

[69] Fernández-Peralbo M.A., Gómez-Gómez E., Calderón-Santiago M., Carrasco-Valiente J., Ruiz-García J., Requena-Tapia M.J., Luque de Castro M.D., Priego-Capote F. (2016). Prostate cancer patients–negative biopsy controls discrimination by untargeted metabolomics analysis of urine by lc-qtof: Upstream information on other omics. Sci. Rep..

[70] Dereziński P., Klupczynska A., Sawicki W., Pałka J.A., Kokot Z.J. (2017). Amino acid profiles of serum and urine in search for prostate cancer biomarkers: A pilot study. Int. J. Med. Sci..

[71] Lopomo A., Coppedè F., Saldanha S. (2018). Chapter 12-Epigenetic signatures in the diagnosis and prognosis of cancer. Epigenetic Mechanisms in Cancer.

[72] Yoruker E.E., Holdenrieder S., Gezer U. (2016). Blood-based biomarkers for diagnosis, prognosis and treatment of colorectal cancer. Clin. Chim. Acta Int. J. Clin. Chem..

[73] Qiu Y., Cai G., Su M., Chen T., Liu Y., Xu Y., Ni Y., Zhao A., Cai S., Xu L.X. (2010). Urinary metabonomic study on colorectal cancer. J. Proteome Res..

[74] Uchiyama K., Yagi N., Mizushima K., Higashimura Y., Hirai Y., Okayama T., Yoshida N., Katada K., Kamada K., Handa O. (2017). Serum metabolomics analysis for early detection of colorectal cancer. J. Gastroenterol..

[75] Nishiumi S., Kobayashi T., Kawana S., Unno Y., Sakai T., Okamoto K., Yamada Y., Sudo K., Yamaji T., Saito Y. (2017). Investigations in the possibility of early detection of colorectal cancer by gas chromatography/triple-quadrupole mass spectrometry. Oncotarget.

[76] Chen J.L., Fan J., Yan L.S., Guo H.Q., Xiong J.J., Ren Y., Hu J.D. (2012). Urine metabolite profiling of human colorectal cancer by capillary electrophoresis mass spectrometry based on mrb. Gastroenterol. Res. Pract..

[77] Cheng Y., Xie G., Chen T., Qiu Y., Zou X., Zheng M., Tan B., Feng B., Dong T., He P. (2012). Distinct urinary metabolic profile of human colorectal cancer. J. Proteome Res..

[78] Chan A.W., Mercier P., Schiller D., Bailey R., Robbins S., Eurich D.T., Sawyer M.B., Broadhurst D. (2016). (1)h-nmr urinary metabolomic profiling for diagnosis of gastric cancer. Br. J. Cancer.

[79] Jung J., Jung Y., Bang E.J., Cho S.I., Jang Y.J., Kwak J.M., Ryu D.H., Park S., Hwang G.S. (2014). Noninvasive diagnosis and evaluation of curative surgery for gastric cancer by using nmr-based metabolomic profiling. Ann. Surg. Oncol..

[80] Kim H., Hwang Y., Sung H., Jang J., Ahn C., Kim S.G., Yoo K.Y., Park S.K. (2018). Effectiveness of gastric cancer screening on gastric cancer incidence and mortality in a community-based prospective cohort. Cancer Res. Treat..

[81] Choi K.S., Suh M. (2014). Screening for gastric cancer: The usefulness of endoscopy. Clin. Endosc..

[82] Dong L.M., Shu X.O., Gao Y.T., Milne G., Ji B.T., Yang G., Li H.L., Rothman N., Zheng W., Chow W.H. (2009). Urinary prostaglandin e2 metabolite and gastric cancer risk in the shanghai women’s health study. Cancer Epidemiol. Biomark. Prev..

[83] Chen J.L., Fan J., Lu X.J. (2014). Ce-ms based on moving reaction boundary method for urinary metabolomic analysis of gastric cancer patients. Electrophoresis.

[84] Chen T., Xie G., Wang X., Fan J., Qiu Y., Zheng X., Qi X., Cao Y., Su M., Wang X. (2011). Serum and urine metabolite profiling reveals potential biomarkers of human hepatocellular carcinoma. Mol. Cell. Proteom..

[85] Bampa G., Moraitou D., Metallidou P., Tsolaki M. (2017). Metacognition in mci: A research proposal on assessing the efficacy of a metacognitive intervention. Hell. J. Nucl. Med..

[86] Seesaard T., Khunarak C., Kerdcharoen T., Kitiyakara T. Development of an electronic nose for detection and discrimination of exhaled breath of hepatocellular carcinoma patients. Proceedings of the 2012 IEEE International Conference on Systems, Man, and Cybernetics (SMC).

[87] Qin T., Liu H., Song Q., Song G., Wang H.Z., Pan Y.Y., Xiong F.X., Gu K.S., Sun G.P., Chen Z.D. (2010). The screening of volatile markers for hepatocellular carcinoma. Cancer Epidemiol. Biomark. Prev..

[88] Amal H., Ding L., Liu B.B., Tisch U., Xu Z.Q., Shi D.Y., Zhao Y., Chen J., Sun R.X., Liu H. (2012). The scent fingerprint of hepatocarcinoma: In-vitro metastasis prediction with volatile organic compounds (vocs). Int. J. Nanomed..

[89] Wu H., Xue R., Dong L., Liu T., Deng C., Zeng H., Shen X. (2009). Metabolomic profiling of human urine in hepatocellular carcinoma patients using gas chromatography/mass spectrometry. Anal. Chim. Acta.

[90] Osman D., Ali O., Obada M., El-Mezayen H., El-Said H. (2017). Chromatographic determination of some biomarkers of liver cirrhosis and hepatocellular carcinoma in egyptian patients. Biomed. Chromatogr. BMC.

[91] Shariff M.I., Kim J.U., Ladep N.G., Crossey M.M., Koomson L.K., Zabron A., Reeves H., Cramp M., Ryder S., Greer S. (2016). Urinary metabotyping of hepatocellular carcinoma in a uk cohort using proton nuclear magnetic resonance spectroscopy. J. Clin. Exp. Hepatol..

[92] Jin X., Yun S.J., Jeong P., Kim I.Y., Kim W.J., Park S. (2014). Diagnosis of bladder cancer and prediction of survival by urinary metabolomics. Oncotarget.

[93] Alberice J.V., Amaral A.F., Armitage E.G., Lorente J.A., Algaba F., Carrilho E., Marquez M., Garcia A., Malats N., Barbas C. (2013). Searching for urine biomarkers of bladder cancer recurrence using a liquid chromatography-mass spectrometry and capillary electrophoresis-mass spectrometry metabolomics approach. J. Chromatogr. A.

[94] Huang Z., Lin L., Gao Y., Chen Y., Yan X., Xing J., Hang W. (2011). Bladder cancer determination via two urinary metabolites: A biomarker pattern approach. Mol. Cell. Proteom..

[95] Nakai Y., Anai S., Onishi S., Masaomi K., Tatsumi Y., Miyake M., Chihara Y., Tanaka N., Hirao Y., Fujimoto K. (2015). Protoporphyrin ix induced by 5-aminolevulinic acid in bladder cancer cells in voided urine can be extracorporeally quantified using a spectrophotometer. Photodiagn. Photodyn. Ther..

[96] Cauchi M., Weber C.M., Bolt B.J., Spratt P.B., Bessant C., Turner D.C., Willis C.M., Britton L.E., Turner C., Morgan G. (2016). Evaluation of gas chromatography mass spectrometry and pattern recognition for the identification of bladder cancer from urine headspace. Anal. Methods.

[97] Capasso M., Franceschi M., Rodriguez-Castro K.I., Crafa P., Cambie G., Miraglia C., Barchi A., Nouvenne A., Leandro G., Meschi T. (2018). Epidemiology and risk factors of pancreatic cancer. Acta Bio Medica Atenei Parm..

[98] Aier I., Semwal R., Sharma A., Varadwaj P.K. (2019). A systematic assessment of statistics, risk factors, and underlying features involved in pancreatic cancer. Cancer Epidemiol..

[99] Chhoda A., Lu L., Clerkin B.M., Risch H., Farrell J.J. (2019). Current approaches to pancreatic cancer screening. Am. J. Pathol..

[100] Long N.P., Yoon S.J., Anh N.H. (2018). A systematic review on metabolomics-based diagnostic biomarker discovery and validation in pancreatic cancer. Metabolomics.

[101] Itoi T., Sugimoto M., Umeda J., Sofuni A., Tsuchiya T., Tsuji S., Tanaka R., Tonozuka R., Honjo M., Moriyasu F. (2017). Serum metabolomic profiles for human pancreatic cancer discrimination. Int. J. Mol. Sci..

[102] Xie G., Lu L., Qiu Y., Ni Q., Zhang W., Gao Y.T., Risch H.A., Yu H., Jia W. (2015). Plasma metabolite biomarkers for the detection of pancreatic cancer. J. Proteome Res..

[103] Takhar A.S., Palaniappan P., Dhingsa R., Lobo D.N. (2004). Recent developments in diagnosis of pancreatic cancer. BMJ.

[104] Nguyen V., Hurton S., Ayloo S., Molinari M. (2015). Advances in pancreatic cancer: The role of metabolomics. J. Pancreas.

[105] Lindahl A., Heuchel R., Forshed J., Lehtio J., Lohr M., Nordstrom A. (2017). Discrimination of pancreatic cancer and pancreatitis by lc-ms metabolomics. Metabolomics.

[106] Fontana A., Copetti M., Di Gangi I.M., Mazza T., Tavano F., Gioffreda D., Mattivi F., Andriulli A., Vrhovsek U., Pazienza V. (2016). Development of a metabolites risk score for one-year mortality risk prediction in pancreatic adenocarcinoma patients. Oncotarget.

[107] Mayers J.R., Wu C., Clish C.B., Kraft P., Torrence M.E., Fiske B.P., Yuan C., Bao Y., Townsend M.K., Tworoger S.S. (2014). Elevation of circulating branched-chain amino acids is an early event in human pancreatic adenocarcinoma development. Nat. Med..

[108] Zou W., She J., Tolstikov V.V. (2013). A comprehensive workflow of mass spectrometry-based untargeted metabolomics in cancer metabolic biomarker discovery using human plasma and urine. Metabolites.

[109] OuYang D., Xu J., Huang H., Chen Z. (2011). Metabolomic profiling of serum from human pancreatic cancer patients using 1 h nmr spectroscopy and principal component analysis. Appl. Biochem. Biotechnol..

[110] Zhang H., Wang Y., Gu X., Zhou J., Yan C. (2011). Metabolomic profiling of human plasma in pancreatic cancer using pressurized capillary electrochromatography. Electrophoresis.

[111] Urayama S., Zou W., Tolstikov V. (2010). Comprehensive mass spectrometry based metabolomic profiling of blood plasma reveals potent discriminatory classifiers of pancreatic adenocarcinoma. Gastroenterology.

[112] Unger K., Mehta K.Y., Kaur P., Wang Y., Menon S.S., Jain S.K., Moonjelly R.A., Suman S., Datta K., Singh R. (2018). Metabolomics based predictive classifier for early detection of pancreatic ductal adenocarcinoma. Oncotarget.

[113] Hori Y., Miyabe K., Yoshida M., Nakazawa T., Hayashi K., Naitoh I., Shimizu S., Kondo H., Nishi Y., Umemura S. (2015). Impact of tp53 codon 72 and mdm2 snp 309 polymorphisms in pancreatic ductal adenocarcinoma. PLoS ONE.

[114] Kaur P., Sheikh K., Kirilyuk A., Kirilyuk K., Ressom H.W., Cheema A.K., Kallakury B. Metabolomic profiling for biomarker discovery in pancreatic cancer. Proceedings of the 2010 IEEE International Conference on Bioinformatics and Biomedicine (BIBM).

[115] Arasaradnam R.P., Wicaksono A., O’Brien H., Kocher H.M., Covington J.A., Crnogorac-Jurcevic T. (2018). Noninvasive diagnosis of pancreatic cancer through detection of volatile organic compounds in urine. Gastroenterology.

[116] Napoli C., Sperandio N., Lawlor R.T., Scarpa A., Molinari H., Assfalg M. (2012). Urine metabolic signature of pancreatic ductal adenocarcinoma by (1)h nuclear magnetic resonance: Identification, mapping, and evolution. J. Proteome Res..

[117] Davis V.W., Schiller D.E., Eurich D., Bathe O.F., Sawyer M.B. (2013). Pancreatic ductal adenocarcinoma is associated with a distinct urinary metabolomic signature. Ann. Surg. Oncol..

[118] Lusczek E.R., Paulo J.A., Saltzman J.R., Kadiyala V., Banks P.A., Beilman G., Conwell D.L. (2013). Urinary 1 h-nmr metabolomics can distinguish pancreatitis patients from healthy controls. JOP.

[119] Radon T.P., Massat N.J., Jones R., Alrawashdeh W., Dumartin L., Ennis D., Duffy S.W., Kocher H.M., Pereira S.P., Guarner L. (2015). Identification of a three-biomarker panel in urine for early detection of pancreatic adenocarcinoma. Clin. Cancer Res..

[120] Mayerle J., Kalthoff H., Reszka R., Kamlage B., Peter E., Schniewind B., González Maldonado S., Pilarsky C., Heidecke C.D., Schatz P. (2018). Metabolic biomarker signature to differentiate pancreatic ductal adenocarcinoma from chronic pancreatitis. Gut.

[121] American Cancer Society Key Statistics about Kidney Cancer. https://www.cancer.org/cancer/kidney-cancer/about/key-statistics.html.

[122] American Cancer Society Tests for Kidney Cancer. https://www.cancer.org/cancer/kidney-cancer/detection-diagnosis-staging/how-diagnosed.html.

[123] Rossi S.H., Klatte T., Usher-Smith J., Stewart G.D. (2018). Epidemiology and screening for renal cancer. World J. Urol..

[124] Kim K., Aronov P., Zakharkin S.O., Anderson D., Perroud B., Thompson I.M., Weiss R.H. (2009). Urine metabolomics analysis for kidney cancer detection and biomarker discovery. Mol. Cell. Proteom..

[125] Rogers M.A., Clarke P., Noble J., Munro N.P., Paul A., Selby P.J., Banks R.E. (2003). Proteomic profiling of urinary proteins in renal cancer by surface enhanced laser desorption ionization and neural-network analysis. Identif. Key Issues Affect. Potential Clin. Util..

[126] Han W.K., Alinani A., Wu C.L., Michaelson D., Loda M., McGovern F.J., Thadhani R., Bonventre J.V. (2005). Human kidney injury molecule-1 is a tissue and urinary tumor marker of renal cell carcinoma. J. Am. Soc. Nephrol..

[127] Bosso N., Chinello C., Picozzi S.C., Gianazza E., Mainini V., Galbusera C., Raimondo F., Perego R., Casellato S., Rocco F. (2008). Human urine biomarkers of renal cell carcinoma evaluated by clinprot. Proteom. Clin. Appl..

[128] Ganti S., Taylor S.L., Kim K., Hoppel C.L., Guo L., Yang J., Evans C., Weiss R.H. (2012). Urinary acylcarnitines are altered in human kidney cancer. Int. J. Cancer.

[129] Baird D.C., Meyers G.J., Hu J.S. (2018). Testicular cancer: Diagnosis and treatment. Am. Fam. Phys..

[130] Neely D., Gray S. (2011). Primary carcinoid tumour of the testis. Ulster Med. J..

[131] American Cancer Society Tests for Testicular Cancer. https://www.cancer.org/cancer/testicular-cancer/detection-diagnosis-staging/how-diagnosed.html.

[132] Milose J.C., Filson C.P., Weizer A.Z., Hafez K.S., Montgomery J.S. (2011). Role of biochemical markers in testicular cancer: Diagnosis, staging, and surveillance. Open Access J. Urol..

[133] Lipsett M.B., Sarfaty G.A., Wilson S.H., Bardin C.W., Fishman L.M. (1966). Metabolism of testosterone and related steroids in metastatic interstitial cell carcinoma of testis. J. Clin. Investig..

[134] Von Eyben F.E. (1978). Biochemical markers in advanced testicular tumours: Serum lactate dehydrogenase, urinary chorionic gonadotropin and total urinary estrogens. Cancer.

[135] Kalyanaraman B. (2017). Teaching the basics of cancer metabolism: Developing antitumor strategies by exploiting the differences between normal and cancer cell metabolism. Redox Biol..

[136] Hsu W.Y., Chen C.J., Huang Y.C., Tsai F.J., Jeng L.B., Lai C.C. (2013). Urinary nucleosides as biomarkers of breast, colon, lung, and gastric cancer in taiwanese. PLoS ONE.

[137] Loeb S., Bjurlin M.A., Nicholson J., Tammela T.L., Penson D.F., Carter H.B., Carroll P., Etzioni R. (2014). Overdiagnosis and overtreatment of prostate cancer. Eur. Urol..

[138] Welch H.G., Black W.C. (2010). Overdiagnosis in cancer. J. Natl. Cancer Inst..

[139] Kalager M., Wieszczy P., Lansdorp-Vogelaar I., Corley D.A., Bretthauer M., Kaminski M.F. (2018). Overdiagnosis in colorectal cancer screening: Time to acknowledge a blind spot. Gastroenterology.

[140] Davies L., Petitti D.B., Martin L., Woo M., Lin J.S. (2018). Defining, estimating, and communicating overdiagnosis in cancer screeningoverdiagnosis in cancer screening. Ann. Intern. Med..

[141] Kim Y., Koo I., Jung B.H., Chung B.C., Lee D. (2010). Multivariate classification of urine metabolome profiles for breast cancer diagnosis. BMC Bioinform..

[142] Rodriguez-Suarez E., Siwy J., Zurbig P., Mischak H. (2014). Urine as a source for clinical proteome analysis: From discovery to clinical application. Biochim. Biophys. Acta.

[143] Struck-Lewicka W., Kordalewska M., Bujak R., Yumba Mpanga A., Markuszewski M., Jacyna J., Matuszewski M., Kaliszan R., Markuszewski M.J. (2015). Urine metabolic fingerprinting using lc-ms and gc-ms reveals metabolite changes in prostate cancer: A pilot study. J. Pharm. Biomed. Anal..

[144] van de Goor R.M.G.E., Leunis N., van Hooren M.R.A., Francisca E., Masclee A., Kremer B., Kross K.W. (2017). Feasibility of electronic nose technology for discriminating between head and neck, bladder, and colon carcinomas. Eur. Arch. Oto Rhino Laryngol..

[145] Khalid T., White P., De Lacy Costello B., Persad R., Ewen R., Johnson E., Probert C.S., Ratcliffe N. (2013). A pilot study combining a gc-sensor device with a statistical model for the identification of bladder cancer from urine headspace. PLoS ONE.

[146] Güntner A.T., Koren V., Chikkadi K., Righettoni M., Pratsinis S.E. (2016). E-nose sensing of low-ppb formaldehyde in gas mixtures at high relative humidity for breath screening of lung cancer?. ACS Sens..

[147] Willis C.M., Church S.M., Guest C.M., Cook W.A., McCarthy N., Bransbury A.J., Church M.R.T., Church J.C.T. (2004). Olfactory detection of human bladder cancer by dogs: Proof of principle study. BMJ.

[148] Horstmann M., Steinbach D., Fischer C., Enkelmann A., Grimm M.O., Voss A. (2015). 834 urine based bladder cancer detection by an electronic nose system: First results of a pilot study. Eur. Urol. Suppl..

[149] Brooks S.W., Moore D.R., Marzouk E.B., Glenn F.R., Hallock R.M. (2015). Canine olfaction and electronic nose detection of volatile organic compounds in the detection of cancer: A review. Cancer Investig..

[150] Nagata Y. (2003). Measurement of Odor Threshold by Triangle Odor Bag Method. International Symposium on Odor Measurement. Asian Network on Odor Measurement and Control.

[151] Van Gemert L.J. (2011). Odour Threshold. Compilation of Odour Threshold Values in Air, Eater and Other Media.

[152] Laska M., Buettner A. (2017). Human and animal olfactory capabilities compared. Springer Handbook of Odor.

[153] Smith T. Can Dogs Smell Cancer?. http://companiontraining.com/can-dogs-smell-cancer.

